# Autophagy regulation by RNA alternative splicing and implications in human diseases

**DOI:** 10.1038/s41467-022-30433-1

**Published:** 2022-05-18

**Authors:** Patricia González-Rodríguez, Daniel J. Klionsky, Bertrand Joseph

**Affiliations:** 1 Institute of Environmental Medicine, Toxicology Unit, Karolinska Institutet, Stockholm, Sweden; 2 Life Sciences Institute, Department of Molecular, Cellular and Developmental Biology, University of Michigan, Ann Arbor, MI USA; 3 Present Address: Division of Biochemistry, Department of Molecular Medicine, Institute of Basic Medical Sciences, University of Oslo, Oslo, Norway

**Keywords:** Autophagy, Alternative splicing

## Abstract

Autophagy and RNA alternative splicing are two evolutionarily conserved processes involved in overlapping physiological and pathological processes. However, the extent of functional connection is not well defined. Here, we consider the role for alternative splicing and generation of autophagy-related gene isoforms in the regulation of autophagy in recent work. The impact of changes to the RNA alternative splicing machinery and production of alternative spliced isoforms on autophagy are reviewed with particular focus on disease relevance. The use of drugs targeting both alternative splicing and autophagy as well as the selective regulation of single autophagy-related protein isoforms, are considered as therapeutic strategies.

## Abstract (web summary)

Both alternative splicing and autophagy are core cell biological processes, but where they intersect has received little attention. Here, the authors reflect on recent connections identified between these pathways and consider their impact on human disease.

## Introduction

In mammals, autophagy is an evolutionarily conserved process where cytoplasmic components are typically degraded within the lysosome. Consequently, the degraded cargo is recycled depending on the requirement of the cells for different anabolic pathways^1,2^. There are multiple forms of autophagy, which differ in the nature of the cargo (from individual proteins to entire organelles), the mechanism of delivery to the degradative compartment (via a transient vesicle or by direct uptake at the limiting membrane of the lysosome) and the function of the process. The best-characterized form of degradative autophagy is termed macroautophagy, referred to as autophagy hereafter.

**Fig. 1:**
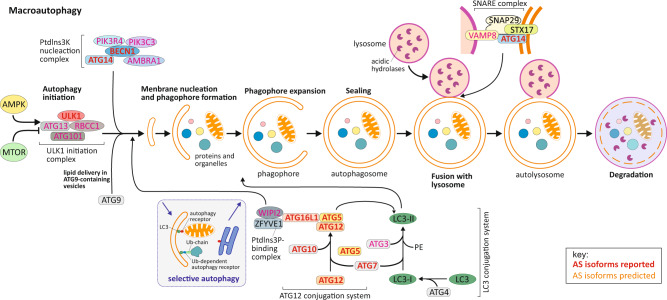
Macroautophagy machinery with indication of components presenting alternative splice isoforms. Bulk autophagy starts with the stepwise engulfment of cytoplasmic material by the phagophore, which matures into a double-layered vesicle named an autophagosome. AMPK and MTOR promote and repress autophagy induction, respectively, through phosphorylation of ULK1 at distinct residues. The ULK1-containing initiation complex triggers phagophore formation by phosphorylating components of the class III Ptdlns3K nucleation complex. The activated Ptdlns3K nucleation complex generates PtdIns3P, which leads to the recruitment of the effector proteins WIPI2 and ZFYVE1/DFCP1. Two Ubl conjugation systems are part of the expansion process. The ATG12-conjugation system that supports in the formation of ATG12–ATG5-ATG16L1 ternary complex, which in turn promotes the second conjugation reaction. The second system, the LC3 conjugation system, involves the conjugation of PE to MAP1LC3/LC3. Lipid conjugation converts the soluble processed form of LC3, named LC3-I, into the phagophore membrane-bound LC3-II form. LC3-II functions in phagophore expansion, and in cargo recognition of ubiquitinated proteins and organelles, including upon selective autophagy with the involvement of autophagy receptors and Ub-dependent autophagy receptors (see Box 2 for further details). As a result of membrane expansion and sealing, the autophagic cargo become sequestered within the mature autophagosome which then fuses with a lysosome. A set of SNARE proteins, are essential for the fusion between autophagosomes and lysosomes. ATG14 promotes the SNARE-mediated autophagosome-lysosome fusion. Docking and fusion of the outer autophagosomal membrane with that of the lysosome exposes the inner vesicle to the lysosomal lumen, where acidic hydrolases degrade and recycle the macromolecular components for cellular use. Key: Component of the autophagic machinery with predicted alternative splice isoforms are highlighted with orange text, and those with reported alternative splice isoforms are highlighted with red bold text.

Autophagy occurs constitutively at a basal level and is induced by various types of stress. Following induction, the process is marked by the generation of a sequestering compartment, the phagophore. During nonselective autophagy, the phagophore membrane may encapsulate random cytoplasm, or regions that have been segregated for example by localized phase separation^3^. In contrast, selective autophagy involves receptors that link the cargo with the phagophore through binding to an Atg8 (autophagy-related 8)-family protein (a list of abbreviations, including gene/protein names, are included in Supplementary Note 1), which is anchored to the phagophore membrane^4^. Expansion and closure of the phagophore generate a complete double-membrane autophagosome, which subsequently fuses with a lysosome to allow exposure of the cargo to the hydrolytic environment; breakdown products are then released through various permeases into the cytoplasm, where they can be used for different anabolic purposes. Bulk, as well as selective, autophagy is a tightly regulated cellular process which includes the involvement of a core set of ATG proteins and other autophagy-related regulators (Fig. 1 and Box 1).

The routine removal of cytoplasm plays an important role in cellular homeostasis. Similarly, the degradation of damaged or superfluous organelles is critical in minimizing oxidative stress and preventing protein and DNA damage. Accordingly, dysfunctional autophagy is associated with a wide array of diseases in humans, including cancer, neurodegeneration, metabolic diseases such as diabetes, and organ-related diseases including those of the heart, lung, liver, muscle, kidney, and eye. Autophagy also plays an important role in the immune response and protection against various microbial infections. To complicate the matter, the presence of autophagy, not just its absence, can be detrimental. For example, some pathogens subvert autophagy to form a replicative niche, whereas certain cancer cells may rely on the cytoprotective functions of autophagy to survive in a nutrient-poor environment, or to resist anti-cancer treatments. Nonetheless, there is tremendous potential in manipulating autophagy for therapeutic purposes.

Box 1 with illustration| Selective autophagic machinery with indication of components presenting alternative splice isoforms

(**a**) Upon mitophagy, mitochondrial autophagy receptors such as BNIP3 and BNIP3L/NIX interact directly with LC3-II on the phagophore. PINK1 accumulates and activates itself on the outer membrane of damaged mitochondria. MFN2 phosphorylated by PINK1 acts as a receptor for PRKN/PARKIN on mitochondria. The polyubiquitination of mitochondrial PRKN substrates lead to their interaction with SQSTM1. The cargo-SQSTM1 complex is then selectively tethered to the autophagosome through interaction of SQSTM1 with LC3-II. (**b**) Reticulophagy leads to the encapsulation of pieces of the ER tubules or ER sheets within autophagosomes. RETREG1/FAM134B, SEC62, and CCPG1 on ER sheets, as well as RTN3L, ATL3 and TEX264 on ER tubules, have been identified as mammalian reticulophagy receptors that all contain LIR motifs allowing them to bind to LC3-II-decorated phagophores. (**c**) Chaperone-mediated autophagy (CMA) involves the selective degradation of KFERQ-like motif-bearing proteins delivered to the lysosomes via chaperone HSPA8/HSC70 and cochaperones, and their internalization in lysosomes via the receptor LAMP2A. Lys-HSPA8, lysosomal HSPA8.

**Key**: Component of the autophagic machinery with predicted alternative splice isoforms are highlighted with orange text, and those with reported alternative splice isoforms are highlighted with red bold text.

### RNA alternative splicing generates protein isoforms for most of the genes encoding components of the core autophagy machinery

**Fig. 2:**
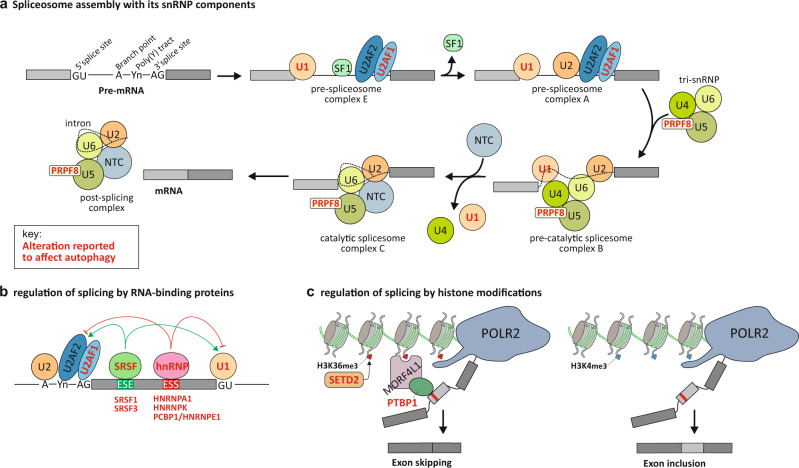
Alternative-splicing machinery with indication of component alterations impacting on autophagy. (**a**) Schematic representation of the stepwise assembly of the spliceosome from its small ribonucleoprotein (snRNP) components. U1 snRNP recognizes and binds to the 5′ splicing site (5′SS), SF1 binds to the branch point site, U2AF2 binds to the polypyrimidine poly(Y) tract and U2AF1 binds to the 3′SS, forming the pre-spliceosome complex E. Next, U2 snRNP replaces SF1 on the branch point site to form the pre-spliceosome complex A. The tri-snRNP, consisting of U5-, U4- and U6 snRNPs, joins complex A to form the pre-catalytic spliceosome complex B. Thereafter, U1 and U4 snRNP leaves, the U6 snRNP binds to the 5′SS, and NTC is recruited so that the U6 snRNP and the U2 snRNP can pair, thereby generating the catalytic spliceosome complex C. Two subsequent transesterification reactions result in the creation of a post-splicing complex with intron exclusion, and the formation of mature mRNA with interconnected exons. (**b**) Regulation of splicing by RNA-binding proteins (RBPs). SRSF-bound exonic splicing enhancer (ESE) can stimulate splicing at up- and downstream splice sites by facilitating the interaction of U2AF or U1 snRNP with the 3′SS and 5′SS, respectively, on the pre-mRNA. Heterogeneous nuclear ribonucleoprotein (hnRNP)-bound exonic splicing silencers (ESS), antagonize the effect of SRSF proteins and suppress the splice sites. (**c**) Regulation of splicing by an epigenetic mechanism such as histone modifications. The recruitment of RBPs can also occur through binding to specific histone marks. For example, SETD2-mediated trimethylation of H3K36 can induce exon skipping by recruiting the splicing factors such as PTBP1 via the chromodomain adaptor protein MORF4L1/MRG15. In contrast, inclusion of exons is increased with H3K4me3 histone modification that reduces MORF4L1 recruitment. Key: Alteration in the alternative-splicing machinery and its regulators reported to have an impact on the autophagic process are highlighted with red bold text.

This perspective focus on protein isoforms produced from a single gene locus though spliceosome-mediated RNA alternative splicing (Box 2). The stepwise assembly of the spliceosome complex with its five U1, U2, U4/U6, and U5 small nuclear ribonucleoproteins (snRNPs) core components is illustrated in Fig. 2. SnRNPs are RNA-protein complexes that consist of a small nuclear RNA (snRNA) and several snRNP-specific proteins including Sm RNA-binding proteins^5^. In fact, the spliceosome machinery encompasses hundreds of proteins including splicing regulators such as serine and arginine rich splicing factor (SRSF) proteins, heterogeneous ribonucleoproteins (HNRNPs), as well as other RNA-binding proteins (RBPs)^6^. Selective components of the spliceosome recognize unique sequences, called 3′ splice-site (3′SS) and 5′SS, within the pre-mRNA to promote alternative splicing. A branch point site is involved in RNA loop formation, which eventually results in the removal of introns and the subsequent re-joining of exons (Fig. 2a). Alternative splicing is also regulated by splicing regulatory elements, *cis-*acting sequencing in pre-mRNA that enhance or silence the splicing of introns^7^. The recruitment of SRSFs and HNRNPs are involved in the regulation of alternative splicing by these *cis-*acting regulatory elements (Fig. 2b). Alternative splicing can occur during transcription, so-called co-transcriptional splicing^8^. This mechanism requires an interplay between the transcriptional and splicing machineries and involves epigenetic mechanisms. For instance, chromatin modifications can modulate POLR2 (RNA polymerase II) elongation, and thereby affect alternative splicing. Epigenetic modification including DNA methylation patterns and specific histone posttranslational modifications (e.g., trimethylation of lysine 36 on histone H3 [H3K36me3] or H3K4me3) are coupled to exon recognition and alternative splicing events (reviewed in ref. ^9^) (Fig. 2c).

Approximately 95% of human multi-exon genes are subject to alternative splicing, catalyzed by the spliceosome^10^. Most translated splice variants are thought to function in closely related biochemical pathways. However, isoforms might exhibit distinct enzymatic activities, subcellular localization, expression, and/or molecular interactions, and may thereby exert different or even opposing effects on multiple biological processes, including autophagy. In fact, a search of the gene databases, as well as a survey of the literature reveal that protein isoforms exist for the bulk of genes encoding components of the autophagy core machinery (Fig. 1 and Supplementary Table 1).

Box 2 with illustration| Protein isoforms, gene isoforms, and mechanism producing them

(**a**) Different variants of a protein, i.e., protein isoforms, can be produced from closely related genes (referred to as a gene family), or from a single gene and in the latter case are then termed as gene isoforms. A gene family, a set of several similar genes located at different loci in the genome, is formed by the duplication of an ancestral gene. In contrast, the so-called gene isoforms are produced from a single gene locus. The transcripts for these protein isoforms may share different levels of identity in different regions of their sequence coding for domains and motifs, responsible for particular function(s) or interaction(s). As a result, the protein isoforms may exhibit identical or similar biological roles, as well as distinctive functions. (**b**) The so-called gene isoforms are produced from the same locus and can differ in their transcription start site/TSS, being the result of alternative promoter usage, differ in their exon usage as a consequence of RNA alternative-splicing event(s), or even exhibit different 3′ ends through preferential usage of distinct polyadenylation/PA sites. In fact, pre-mRNAs can undergo one of multiple forms of alternative splicing such as switching between alternative 5′ and 3′ splice sites, inclusion or skipping of individual “cassette” exons, mutually exclusive splicing of adjacent exons, differential intron retention, and/or other, more complex patterns of splice-site selection^166^.

### Alterations in the RNA alternative splicing regulatory machinery affect autophagy

Alterations in the spliceosome core components, including snRNPs as well as splicing regulatory factors such as SRSF proteins, HNRNPs, other RBPs and chromatin modifiers are reported to contribute to various diseases^11^. Remarkably, many of these alterations in the alternative-splicing machinery are linked to the differential expression of autophagy-related gene splice isoforms and as a result impaired autophagy.

Alterations in major players in the assembly and functions of spliceosome (Fig. 2a), such as *RNU1-1/U1* snRNA (we refer to these snRNA genes by their common names hereafter) within the U1 snRNP complex, PRPF8 (pre-mRNA processing factor 8) within the U5 snRNP complex, or the U2AF1 (U2 small nuclear RNA auxiliary factor 1) subunit of the U2AF complex are all linked to impaired autophagy.

The *U1* snRNA, that serves as the backbone of the **U1 snRNP** complex, but not *U2*, *U4*, *U5*, and *U6* snRNA, is enriched in familial Alzheimer disease (AD), which is characterized by β-amyloid aggregates and MAPT/tau protein hyperphosphorylation, as well as mitochondrial dysfunction and defective mitophagy (reviewed in^12^). Autosomal dominant mutations in *PSEN1* (presenilin 1) and *APP* (amyloid beta precursor protein) genes are the most common cause of early-onset familial AD^13^. The level of *U1* snRNA is considerably higher in PSEN1 APP double-transgenic mice as compared to control littermates^14^. PSEN1 regulates V-ATPase-mediated lysosome acidification^15^, and *PSEN1* mutations are reported to inhibit lysosome acidification. *PSEN1* mutations lead to *U1 snRNA* overexpression, accompanied by its nuclear aggregation, aberrant recruitment to nascent transcripts and RNA splicing^14,16^. Also worth noting, *U1 snRNA* overexpression affects autophagy by an impairment of lysosomal biogenesis and autophagosome-lysosome fusion, which in turn affects the turnover of these peptides and accelerates neuronal cell death^17^. Of note, patients with Down syndrome (DS) have a significantly higher risk of developing early-onset AD, largely owing to a triplication of the *APP* gene, located on chromosome 21. Remarkably, the U1 snRNP proteopathy, the deposition of both amyloid plaques and neurofibrillary tangles, and mitochondrial dysfunction as well as alteration of mitophagy, are reported in patients with DS^18,19^. MTOR (mechanistic target of rapamycin kinase) hyperactivation in DS appears to cause the deficits in autophagy induction, autophagosome formation, and mitophagy^19^. Hence, targeting autophagy for the treatment of both AD and DS has been proposed^19,20^. To date, the suggested interventions do not consider the alteration in the alternative-splicing machinery that drive the autophagy deficiency, and how mitochondria dysfunction is triggered in these patients.

The pre-mRNA splicing factor **PRPF8** is a component of the U5 snRNP, a central module of the spliceosome^21^. An in vitro RNA interference screen identified PRPF8, as an essential mediator of hypoxia-induced mitophagy. Indeed, *PRPF8* knockdown leads to reduced mitophagosome formation and mitochondrial clearance. *PRPF8*-deficiency is associated with aberrant mRNA splicing of *ULK1* (unc-51 like autophagy activating kinase 1), with enhanced skipping of exon 22 and exons 22-23^22^.

**U2AF1/U2AF35** heterodimerizes with U2AF2/U2AF65 to form the pre-mRNA splicing factor U2AF, which plays a role in defining the functional 3′SS in pre-mRNA^23^. Recurrent somatic mutations in U2AF1 are identified in multiple cancer types^24,25^. Heterozygous substitution of residue Ser34, *U2AF1*^*S34F*^, is a frequently observed mutation. In pro-B Ba/F3 cells, expression of U2AF1^S34F^ mutant protein causes the preferential use of distal cleavage and polyadenylation sites, and thereby alteration of mRNA 3′ end formation, on numerous mRNAs including *ATG7*. In the presence of an aberrant long 3′UTR, the *ATG7* mRNA is translated with low efficiency, resulting in reduced ATG7 protein levels. This is a direct consequence of the U2AF1^S34F^ mutant protein, as normal ATG7 expression can be re-established upon suppression of its expression. *U2AF1*^*S34F*^-expressing Ba/F3 cells, like Ba/F3 cells expressing an *ATG7-*targeting shRNA, exhibit a lower LC3B-II:LC3B-I ratio but higher SQSTM1/p62 (sequestosome 1) expression levels as compared to control Ba/F3-V cells, indicative of reduced autophagy.

Alterations in RNA-binding proteins and regulators of RNA alternative splicing (Fig. 2b), including SRSF proteins, HNRNPs, as well as other RBPs have also been associated with deficient autophagy.

**SRSF1 and SRSF3** expression levels are reported to be decreased in various cell types upon autophagy induction^26^. Gain- and loss-of-function studies in oral squamous cell carcinoma reveal that SRSF3 knockdown promotes autophagy induction, whereas its overexpression inhibits this process and promotes tumorigenesis. SRSF3 inhibits the expression of the transcription factors RELA/p65 (RELA proto-oncogene, NF-kB subunit) and FOXO1 (forkhead box O1), and in turn of their downstream target gene *BECN1* (beclin 1) Similarly, in lung adenocarcinoma cells, SRSF1 inhibits autophagosome formation by targeting the BECN1-containing complex^26^. SRSF1 regulates the alternative splicing of *BCL2L1/BCL-xL* (BCL2 like 1), promoting the expression of the long isoform BCL2L1/xL that represses autophagy through interaction with BECN1. In contrast, reduced SRSF1 expression promotes the production of the short isoform BCL2L1/xS that abolishes the interaction with BECN1 and promotes autophagy. In addition, SRSF1 directly interacts with PtdIns3K to disrupt the association of BECN1 with the PtdIns3K complex^26^. Thus, both SRSF1 and SRSF3 could be considered as autophagy suppressors, playing a role in tumorigenesis^26–33^.

Among the hnRNP family members, **HNRNPA1, HNRNPK and PCBP1/HNRNPE1** (poly(rC) binding protein 1) proteins have been associated with the regulation of autophagy in homeostasis and disease. Downregulation of HNRNPA1 is linked to autophagy inhibition and cell death induction^34^. HNRNPA1 binds to the 3′UTR of *BECN1* mRNA and regulates its expression. Increased HNRNPA1 expression is found in multiple cancer types, and positively correlates with BECN1 expression, and is proposed to mediate cancer development. However, whether HNRNPA1-mediated BECN1 expression has a functional impact on autophagy remains to be established^35^.

The observed enhanced HNRNPK expression in acute myeloid leukemia (AML) patients that develop drug resistance to treatment is associated with an increased basal level of autophagy, suggesting that HNRNPK might regulate the expression or alternative splicing of autophagy-related genes^36^. The acetylation of Lys40 on TUBA/α-tubulin is controlled by the opposite actions of HDAC6 (histone deacetylase 6) and ATAT1/α-TAT (alpha tubulin acetryltransferase 1), two enzymes essential for proper microtubule function in mature osteoclasts^37^. Hence HNRNPK downregulates *HDAC6* mRNA expression, which in turn could control microtubule dynamics, required for autophagy completion. In fact, HNRNPK-mediated regulation of HDAC6 is coupled with reduced basal autophagy^38^. PCBP1/HNRNPE1 is also reported to regulate autophagy through MAP1LC3/LC3 (microtubule associated protein 1 light chain 3) downregulation^36^.

Exon Ontology allows the characterization of proteins regulated by alternative splicing. When applied to mesenchymal and epithelial breast cancer cells, the analysis revealed that the splicing factors **MBNL1**, **MBNL2** and **RBFOX2** (RNA-binding fox-1 homolog 2) interact with autophagic components including RUBCN/Rubicon (rubicon autophagy regulator; a BECN1-interacting protein and inhibitor of PtdIns3K activity)^39^ and WDFY3/ALFY (WD repeat And FYVE domain containing 3; a scaffold protein for selective autophagy)^40^. MBNLs are RBPs that regulate alternative-splicing events including exon inclusion and skipping events. RBFOX2 contain an RNA-recognition motif that specifically recognizes the UGCAUG consensus sequence in the introns that flank target exons and promote exon skipping or inclusion depending on whether RBFOX2 binds upstream or downstream of the cassette^41–44^. The absence of *MBNL1*, *MBNL2* and *RBFOX2* promotes exon 14 skipping in *RUBCN* and exon 46 skipping in *WDFY3* that lead to autophagy induction^45^. Interestingly, the manipulation of *RUBCN* splicing results in the decrease of MBNL1 and MBNL2 protein levels, suggesting a feedback loop where the manipulation of splicing factors affect autophagy, and sequentially affect the expression of these same splicing factors. The latter highlights that components of the autophagy machinery could as well have an impact on the expression and dynamics of splicing factors.

Analysis of gene expression profiles from breast, lung and ovarian cancer datasets revealed that genes encoding components of the core spliceosome machinery are overexpressed in malignant tissues as compared to benign ones^46^.

Among the upregulated spliceosomal components, **SNRPE/SmE** (small nuclear ribonucleoprotein polypeptide E) and **SNRPD1/SmD1** (small nuclear ribonucleoprotein D1 polypeptide), are significantly differentially expressed in the above-named cancer types. Knockdown of their expression in breast, lung and melanoma cancer cell lines leads to an increased LC3 lipidation, followed by a marked reduction of cell viability, whereas the latter has little effect on the survival of the non-malignant breast epithelial cells. In the SNRPE-depleted cells a robust decrease in *MTOR* mRNA expression and protein levels is observed, which could explain the observed SNRPE-dependent induction of autophagy. Interestingly, as with SRSF1, this study shows the importance of a proper regulation of the spliceosome-MTOR axis as target to control autophagy-mediated cell death^29,46^.

Deficiencies in epigenetic regulators of RNA alternative splicing (Fig. 2c), such as histone modifying enzymes or protein complexes recognizing the corresponding posttranslational modifications to mediate control of exon splicing are reported to affect autophagy.

**SETD2** (SET domain containing 2, histone lysine methyltransferase) histone methyltransferase mediated-H3K36me3 promotes the recruitment and interaction of the splicing regulator PTBP1/HNRNP1 (polypyrimidine tract binding protein 1) with MORF4L1/MRG15 (mortality factor 4 like 1), an H3K36me3-binding protein in introns of genes transcriptionally active, resulting in alternative splicing^47^. Inactivating mutations in the *SETD2* gene are a frequent molecular feature in clear cell renal cell carcinoma (ccRCC)^48^. Altering *SETD2* gene expression levels is enough to influence the inclusion of exons in genes that are alternatively spliced^49^. H3K36me3 levels differ based on exon utilization, with alternatively spliced exons having lower levels of this histone mark than those that are constitutively included^50,51^. Loss of SETD2 is expected to cause widespread RNA processing defects, and thereby have an impact on many biological processes including autophagy^52^. SETD2 deficiency in ccRCC cells is associated with an aberrant accumulation of both free ATG12 and of an additional ATG12-containing complex, distinct from the ATG12–ATG5 conjugate. Rescue of SETD2 functions in the SETD2-deficient ccRCC cells, or reduction of SETD2 expression level in cells wild type for this enzyme, demonstrate that SETD2 deficiency in ccRCC is directly involved in the acquisition of these alterations in the autophagic process. Hence, SETD2 deficiency is associated with a defect in the ATG12-dependent conjugation system and a decreased autophagic flux, in accord with the role for this ubiquitin (Ub)-like (Ubl) protein conjugation system in autophagosome formation and expansion^53^. Direct interactions of SETD2 and PTBP1 with HNRNPL mediate the crosstalk between histone posttranslational modification, transcription, and pre-mRNA processing in the regulation of alternative splicing^54^. These studies suggest that SETD2-mediated H3K36me3 on its own defines exons rather than the frequency of alternative splicing, because other players such as an H3K36me3-recognizing complex and hnRNPs are required for the splicing event to occur.

**PTBP1** dysregulation has been linked to the onset and development of prostate and ovarian cancer, whereas PTBP1 overexpression is associated with the aggressiveness of glioma, colorectal and breast cancer^55–57^. In the latter, increased PTBP1 expression is linked to the activation of the PTEN (phosphatase and tensin homolog)-AKT signaling pathway and inhibition of autophagy. In contrast, knockdown of PTBP1 expression promotes autophagy and reduce breast cancer cell growth in vivo^56^. In addition, PTPB1 interacts with *ATG10* pre-mRNA via its 3′ UTR, and downregulates its expression level in colorectal cancer cells. PTPB1-mediated ATG10 downregulation increases the expression of epithelial-to-mesenchymal transition proteins and promotes cell migration and invasion^58^. However, whether this mechanism is autophagy-dependent remains to be elucidated.

In general, it should be noted that whereas a few studies report the direct interaction of RNA-binding proteins (e.g., Pat1, and ELAVL4/HuD) with the mRNAs of *ATG* genes, which thereby affect their expression levels, studies reporting direct interactions between the above listed splice factors and the mRNAs of autophagy-related genes that would affect their alternative splicing are missing^59,60^. The use of protein-centric methods, such as cross-linking immunoprecipitation (CLIP), in which a splice factor of interest is cross-linked to, and immunoprecipitated along with its RNA-binding partners, would allow for identification of specifically bound RNAs by sequencing, including autophagy-related genes, that could be of interest in uncovering potential mechanism of action^61^. In addition, because the splicing machinery affects many genes, it is difficult to formally implicate a specific autophagy gene as the causal determinant of an autophagy defect resulting from alterations in splicing machinery. Nevertheless, collectively these studies provide compelling evidence that alterations in the effector as well as regulatory components of the RNA alternative-splicing machinery are associated with impairment of the autophagy process. However, the exact contribution of autophagy deficiency to the observed biological and disease-related effects in the context of an impaired alternative-splicing machinery remain to be fully established.

The above section intentionally focuses on how alterations in the splicing machinery affect autophagy. However, it is worth pointing out that alternative splicing is in turn regulated by autophagy with the autophagic degradation of some splicing factors^62–66^.

### Alternative spliced isoforms of autophagy-related genes differentially affect the autophagic process

**Fig. 3:**
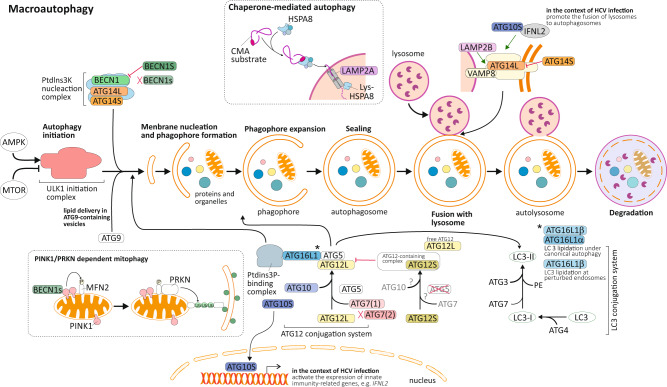
Impact of alternatively spliced autophagy isoforms on autophagic process. BECN1 as a subunit of the class III PtdIns3K nucleation complex contributes to autophagy initiation. A splice isoform, BECN1S, acts a negative regulator of autophagy induction. An additional splice variant, BECN1s, lacks function in the initiation of autophagy but supports PINK1- and PRKN/PARKIN-dependent mitophagy. A long and a short (lacking the homodimerization domain) splice isoform of ATG14 are reported. Whereas both isoforms can promote autophagy induction, they exert opposite effects on the regulation of autophagosome-lysosome fusion, with ATG14L endorsing but ATG14S restraining the formation of autolysosomes. Multiple components of the ATG12-conjugation system present splice isoforms. The presence of ATG12S, a short isoform of ATG12 which appears to be lacking the ability to form a covalent complex with ATG5, leads to the appearance of additional ATG12-containing complexes, beyond the ATG12–ATG5 complex, and the occurrence of free ATG12L, as well as reduced LC3 conjugation and autophagy flux. The ATG16L1 splice isoforms α and β, both allow the recruitment of the ATG12–ATG5 conjugate to the phagophore leading to the subsequent conjugation of LC3 proteins. The ATG16L1β isoform, with its unique lipid-binding region domain, also exerts an additional effect on LC3 lipidation at perturbed endosomes. In the context of hepatitis C virus (HCV) infection, a short ATG10 isoform, nuclear ATG10S, acting as a transcription factor, activates *IFNL2/IL28A* gene expression; IFNL2 protein in turn together with cytoplasmic ATG12S enhances autophagosome-lysosome fusion. ATG7(2), a short splice isoform of the canonical ATG7(1), lacks the ability to bind to LC3 and promote its lipidation. *LAMP2* codes for splice variants that differ in their transmembrane and cytosolic regions within the C terminus region and exert distinct functions. Whereas LAMP2A controls the translocation of chaperone-mediated autophagy substrates across the lysosomal membrane, LAMP2B and LAMP2C promote the formation of autolysosomes or contribute to selective types of autophagy.

Alternative splice isoforms are reported or in silico predicted for autophagy-related genes at each of the steps of this biological process, from initiation to completion. In addition, compelling evidence indicates that splice isoforms for a single autophagy-related gene can either affect the nonselective or selective autophagy machinery at different steps or even exert opposite functions, adding an additional level of complexity in the understanding of the regulation of autophagy (Fig. 3).

**BECN1** as a subunit of the PtdIns3K complex mediates autophagy initiation^67^. DNA sequencing of AML cells revealed the existence of a 3′ alternative splice BECN1 variant (hereafter referred as BECN1S), skipping the last exon of the gene (exon 11) resulting in a truncated C terminus. Overexpression of BECN1S reduces starvation-induced autophagy in AML cells, suggesting that this splice variant might function as a negative regulator of autophagy^68^. An additional BECN1 splice variant that lacks both exon 10 and 11, and designated BECN1 short isoform (BECN1s) has been reported in multiple cell types. Whereas BECN1s is unable to initiate macroautophagy, its expression supports mitophagy. Further investigation showed that BECN1s selectively associates with the outer mitochondrial membrane and induces mitophagy in a PINK1 (PTEN induced kinase 1)-PRKN/PARKIN (parkin RBR E3 ubiquitin protein ligase)-dependent manner^69^. The C terminus of BECN1 contains two domains that can be potentially be affected by this exon skipping: an evolutionarily conserved domain (ECD), involved in interaction with polyQ-domain containing proteins such as ATXN3;^70^ and a domain required for membrane-association^71^. Thus, upon specific stimuli, components of the alternative-splicing machinery can promote the generation of different BECN1 variants that exhibit distinct functions, promoting (BECN1) or inhibiting (BECN1S) nonselective autophagy or selectively promoting (BECN1s) mitophagy.

**ATG14**, like BECN1, is an essential core component of the PtdIns3K nucleation complex involved in early steps of autophagy^72^. Moreover, ATG14 is reported to be involved in the control of membrane tethering and autophagosome-lysosome fusion to form the autolysosome^73^. During the latter, oligomeric ATG14 binds to the soluble N-ethylmaleimide-sensitive factor attachment protein receptor (SNARE) core domain of STX17 (syntaxin 17) through its coiled-coil domain (CCD),stabilizes the STX17-SNAP29 (synaptosome associated protein 29) binary t-SNARE complex on the autophagosomes and primes it for interaction with VAMP8 (vesicle associated membrane protein 8)^74^. Both STX17 binding, and membrane-tethering and fusion-enhancing activities of ATG14 require its evolutionarily conserved Cys repeats at the oligomerization site localized in the N terminus of ATG14. In cells expressing a mutant version of ATG14 lacking the homo-oligomerization site, autophagosomes still efficiently form but their fusion with lysosomes is blocked^73^. Genome databases reveal that the *ATG14* gene encodes a long and a short splice isoform, named ATG14L and ATG14S. Interestingly, the cysteine repeats-containing domain required for ATG14 homo-oligomerization and thereby the promotion of autophagosome-endolysosome fusion is not present in ATG14S, suggesting that only ATG14L can promote this secondary function. In contrast, because this oligomerization domain is not required for the function of ATG14 in the PtdIns3K nucleation complex, both isoforms should promote the formation of phagophores (Fig. 3). Although both isoforms carry distinct roles on the regulation of autophagy, further investigations that aim to elucidate the regulatory-mediated mechanisms of these isoforms and their physio-pathological roles are required.

The **WIPI-family** includes four members**:** WIPI1 (WD repeat domain, phosphoinositide interacting 1), WIPI2, WDR45B/WIPI3 (WD repeat domain 45B) and WDR45/WIPI4. These are gene homologs originated from gene duplication that share functional domains. WIPI1 and WIP2 proteins are recognized for their phosphatidylinositol-3-phosphate (PtdIns3P)-dependent function in autophagy, which is the recruitment of the ATG12–ATG5-ATG16L1 (autophagy-related 16 like 1) complex to nascent autophagosomes. Upon autophagy induction and AMP-activated protein kinase (AMPK) activation, WDR45B- and WDR45-containing complexes translocate to and support further maturation of the autophagosomes^75,76^. However, some WIPI-family members present different splice isoforms that could carry out specific functions in the autophagy process. WIPI1 exhibits two splice isoforms: WIPI1α, exhibiting low binding affinity for ATG16L1 and WIPI1β not yet characterized. WIPI2 contains 5 splice isoforms (A to E), among them only WIPI2B and WIPI2D isoforms are reported to act as PtdIns3P effectors at nascent autophagosomes. However, only WIPI2B binds to ZFYVE1/DFCP1 (zinc finger FYVE-type containing 1) as well as ATG16L1 to be recruited to the ATG12–ATG5-ATG16L1 complex^77^. Of note, WIPI1α binds in lesser degree to ATG16L1 compared to WIPI2B, a mechanism that still needs to be elucidated^78^. Conversely, WDR45B has no reported isoforms, whereas WDR45 presents 3 splice isoforms (WIPI4a, WIPI4b, and WIPI4), but the potentially distinct function(s) of these isoforms remains to be determined.

**ATG10** E2-like enzyme promotes the formation of the ATG12–ATG5 complex and thereby autophagosome formation^79^. ATG10 function plays an essential role in the proliferation and invasion of tumor cells, infection, and inflammation^58,80^. Two distinct isoforms of ATG10 have been reported: *ATG10* encodes the full-length isoform, whereas the *ATG10* short (*ATG10S*) product lack 36 amino acids at the N terminus, encoded by exon 4. Of note, both isoforms have distinct effects on replication after hepatitis C virus (HCV) infection. ATG10S promotes a complete autophagy process that allows the formation of autolysosomes and degradation of the HCV subgenomic and genomic replicons, whereas ATG10 facilitates genomic and subgenomic replicon amplification by promoting autophagosome formation. Moreover, ATG10 lead to the accumulation of autophagosomes in the cell periphery and decreased autophagy flux after HCV infection^81,82^. It appears that ATG10S lacks a Cys44 residue located within the sequence encoded by exon 4. A potential disulfide bond generated between Cys44 and Cys135 seem important for ATG10 function, by allowing a conformational change that leads to inhibition of HCV genomic replication by promoting complete autophagy. Interestingly, the absence of Cys44 in ATG10S appears to be key for its translocation into the nucleus and recruitment to the promoter of *IFNL2/IL28A* (interferon lambda 2), a gene that codes for a cytokine responsible for controlling viral infections and replication but that also might mediate the fusion of the autophagosome and lysosome via direct interaction with ATG10S^82^. Of note, binding to the *IFNL2* promoter occurs in competition with IRF1, which binds at the same core motif. Therefore, ATG10S can display a non-autophagic function as a potent host-antiviral factor but also an autophagic function by rescuing impaired autophagy flux^82^.

The crystal structure of the human ATG12–ATG5 conjugate bound to ATG16L1, a factor required for the recruitment of this conjugate to phagophore membranes, revealed that residues in the C-terminal tail of **ATG12** are required for its interaction with ATG5. This contact interface encompasses the highly conserved residues Phe108, Asp113 and Gly140 of ATG12^83^. The existence of two distinct ATG12 splice isoforms, a canonical long isoform (ATG12L) and a short isoform (ATG12S) is reported in ccRCC cells, with SETD2 deficiency in those cells promoting ATG12S expression^53^. Whereas the transcript for ATG12L originates from four distinct exons, the transcript for ATG12S, only derives from three exons, including an alternative exon 2. ATG12S appears to be missing the carboxyl tail present in ATG12L including the Gly140 residue required for the formation of a covalent complex with ATG5^83^. The presence of the ATG12S isoform in SETD2-deficient ccRCC cells is associated with the presence of an additional ATG12-containing complex, and of free ATG12, which appears based on its molecular weight to be ATG12L, suggesting that ATG12S may compete with ATG12L and thereby act as repressor for the ATG12-conjugation system.

**ATG16L1** allows the recruitment of the ATG12–ATG5 conjugate to the phagophore leading to the subsequent conjugation of LC3 proteins^84^. In addition, ATG16L1 is an interacting partner for the small GTPase RAB33B (RAB33B, member RAS oncogene family), which is involved in vesicle formation and trafficking. It is hypothesized that the interaction between RAB33B and ATG16L1 can act to facilitate autophagosome-lysosome fusion^85^. Beyond their role in promoting autophagosome biogenesis, the ATG16L1-containing complexes have a reported role in the regulation of single-membrane Atg8-family protein conjugation (SMAC) and LC3-associated phagocytosis (LAP)^85,86^. SMAC occurs on pre-formed vesicles such as entotic bodies, phagocytosed bacteria and perturbed endosomes, whereas LAP is involved during phagosome formation. These various functions require distinct domains within ATG16L1, an N-terminal region containing both an ATG5-binding domain and a domain required for LC3 lipidation, a middle region containing a CCD contributing to protein interaction and thereby mediating its homodimerization and interaction with RAB33B, and a C-terminal region with seven WD40 domains dispensable for autophagy but contributing to SMAC and LAP (reviewed in ref. ^87^). At least seven human ATG16L1 splicing isoforms have been suggested in the literature^88^. However, only three of them have been cloned, ATG16L1α (lacking exon 8 and 9), ATG16L1β (lacking exon 9), and ATG16L1γ (including all 20 exons of the gene)^87,89^. Whereas the expression of ATG16L1β was found to colocalize with autophagic vesicles, lysosomes, and mitochondria, ATG16L1α was not found to colocalize with mitochondria^88^. These splice isoforms exhibit distinct subcellular localizations in addition to different tissue-specific patterns and their functions still remain unclear. ATG16L1β distinguishes itself from the other ATG16L1 isoforms by the presence in its sequence of a so-called β-isoform lipid-binding region, located downstream of the CCD, that is required for PtdIns3P-independent SMAC. ATG16L1α which lacks this β-isoform specific region is not able to support LC3 lipidation during endosomal stress^90^.

***ATG16L2***, homolog of *ATG16L1*, also encodes splice variants; a short isoform lacking exon 8, and a long isoform that contains all 18 exons. ATG16L2 shares properties with ATG16L1 including its binding to ATG5, but a weaker binding affinity for RAB33B as compared to ATG16L1. Moreover, despite forming a complex with the ATG12–ATG5 conjugate, ATG16L2 is not essential for phagophore formation. It is speculated that the ability of ATG16L2 to form an ATG12–ATG5-ATG16L2 complex as well as hetero-oligomerize with ATG16L1 could modulate autophagy efficiency^91^. In fact, it appears that ATG16L2, which is overexpressed in several cancers relative to ATG16L1, impairs the conjugation process by competing with ATG16L1 for binding to ATG5^92^. ATG16L1 and ATG16L2 isoforms can participate in distinct types of autophagy^93^. However, the respective roles for ATG16L1 and ATG16L2 splice isoforms remain to be fully elucidated.

**ATG7** E1-like enzyme acts as a central regulator for the two Ubl conjugation systems that are required for LC3/GABARAP (GABA type A receptor-associated protein) lipidation. Three distinct splice isoforms exist in human: ATG7(1) appears to be highly expressed in most human tissues, ATG7(2) exhibits tissue-specific and lower expression, and ATG7(3) is not found to be expressed. ATG7(1), represent the full-length isoform, whereas ATG7(2) is a shorter isoform lacking an exon of 27 amino acids in the C terminus of the protein. Of note, whereas ATG7(2) contains the Cys572 residue involved in bond formation with LC3/GABARAP and ATG12, it lacks the region that promotes homodimerization and the binding between ATG7 and LC3/GABARAP. Therefore, the absence of this region in ATG7(2) implies that this isoform lacks the ability to bind to LC3 and promote its lipidation, a feature only found in ATG7(1)^94,95^. However, independently of this region, ATG7 exerts non-autophagy functions, including the regulation of cell death and the cell cycle by direct binding and regulation of TP53 activity, suggesting that ATG7(2) could be involved in the regulation of these other functions. In addition, ATG7 is identified as a cancer susceptibility gene for cholangiocarcinoma. An *ATG7* germline mutation (p.Arg659*) is reported in affected family members. In addition, an *ATG7* allele variant (p.Asp522Glu) associated with increased risk of cholangiocarcinoma has also been identified in the Icelandic population. In MMNK-1 *ATG7*-null cells, expression of the ATG7(1) isoform or ATG7(p.Asp522Glu) enables LC3 lipidation. In comparison, expression of the ATG7(2) isoform or ATG7(p.Arg659*) is unable to promote LC3-I to LC3-II conversion^94^.

The ***Atg8*** **family** includes 7 gene homologs: *MAP1LC3A*, *MAP1LC3B*, *MAP1LC3B2*, *MAP1LC3C*, *GABARAP*, *GABARAPL1* and *GABARAPL2*. Splice variants have been identified for *MAP1LC3A*, *MAP1LC3B*, *GABARAP* and *GABARAPL1*. Two distinct MAP1LC3A isoforms exist, MAP1LC3A-a (canonical variant) and MAP1LC3A-b (containing one additional exon). The MAP1LC3B-a splice isoform, produced via alternative splicing at so-called NAGNAG splice sites of intron 3, results in the loss of a single amino acid (Arg68), located on the α3 helix of the Ub fold at the C terminus. The MAP1LC3B-a isoform can undergo posttranslational modification by conjugation with the phosphatidylethanolamine (PE) group. However, Arg68 absence in this splice variant, results in conformational changes that inhibit its interaction with and cleavage by ATG4B, a processing step needed to prime the protein for conjugation. Hence, the MAP1LC3B-a isoform should be associated with reduced autophagy flux. Likewise, the GABARAP-a and GABARAPL1-a splice isoforms produced using different 3’ end points for the transcript, lead to the absence of the Gly120 residue, which is essential for the binding and C-terminal cleavage by ATG4 and thus the ability to undergo posttranslational modifications and localization on the phagophore.

**LAMP2** (lysosomal associated membrane protein 2) is one of the most abundant lysosomal membrane components. LAMP2 encodes three splice variants that differ in their transmembrane and cytosolic regions within the C terminus due to the use of 3 alternative exon 9 segments, suggesting that each one might play different cellular functions^96^. Moreover, these isoforms are found in different tissues throughout the body. LAMP2A is located primarily in the placenta, lung and liver; LAMP2B is the main isoform found in the heart and the muscles; LAMP2C is highly expressed in neurons. LAMP2A is the only LAMP2 isoform required for translocation of chaperone-mediated autophagy (CMA) substrates into the lysosome^97^. Instead, LAMP2B and LAMP2C isoforms are likely involved in macroautophagy^98^. The LAMP2B CCD promotes interaction with ATG14 and VAMP8 to promote autophagosome fusion with late endosomes/lysosomes. Interestingly, LAMP2C is reported to interact with RBPs and nucleic acid proteins such as histone 1, suggesting a role in the uptake and degradation of RNA and DNA molecules within the lysosome, processes known as DNautophagy and RNautophagy^99^. LAMP2C also acts as an endogenous negative regulator of CMA counteracting the effect of LAMP2A^96^.

Both **PRKN** and **PINK1** proteins function in a common mitochondrial quality control pathway, whereby disruption of the mitochondrial membrane potential leads to PINK1 stabilization at the mitochondrial outer surface. PINK1 accumulation leads to the recruitment of cytosolic PRKN, which in turn promotes the degradation of the damaged mitochondria by mitophagy. Hence, the PINK1-PRKN-dependent mitophagy signaling pathway is essential for the regulation and protection from stress-mediated mitochondrial dysfunction that eventually results in the loss of dopaminergic neurons in the substantia nigra^100^. PINK1 has two isoforms, the mitochondria-localized full-length isoform PINK1FL and the cytoplasm-localized short isoform PINK1-cyto. Studies suggest that PINK1FL can selectively accumulate at the surface of damaged mitochondria and cooperate with PRKN to trigger the ubiquitination of mitochondrial proteins, which marks mitochondria for degradation via mitophagy^101,102^. In contrast, PINK1-cyto tends to accumulate in the cytosol during proteasomal stress and acts as a regulator of aggresome formation. PINK1-cyto exerts its effect through phosphorylation of the Ub-binding protein SQSTM1, increasing its ability to sequester polyubiquitinated proteins into aggresomes^103^. To date, the existence of 21 human PRKN splice variants, with different expression in tissues and cells, and distinct domain composition, and thus potentially functions, have been suggested^104^. In fact, induction of autophagy in glioma cells upon starvation or treatment with a mitochondrial uncoupling agent, leads to the selective upregulation of three PRKN isoforms, H1, H2 and H20, that happen to be the only three isoforms that contain the N-terminal Ubl domain and the two C-terminal RING domains (RING1, RING2) separated by an “in-between RING”/IBR domain of PRKN^104^. However, it is unknown if the function of PRKN in the regulation of mitophagy, and other biological processes, is mediated by a single protein or by different isoforms.

The endoplasmic reticulum (ER) is the most abundant membrane structure in the cell, which regulates protein synthesis and posttranslational modifications, lipid metabolism and calcium homeostasis^105^. Due to the importance of this organelle, a checkpoint exists that recognizes terminally misfolded proteins and promotes their degradation via essential pathways including ER autophagy-mediated degradation, known as reticulophagy^106^. Reticulophagy requires the function of ER-resident membrane proteins including **RETREG1/FAM134B** (reticulophagy regulator 1), and **RTN3** (reticulon 3) (reviewed in ref. ^107^) (Fig. 1c). RETREG1 is an ER-anchored protein preferentially located in ER sheets, that mediates ER remodeling and scission into autophagosomes by direct binding with MAP1LC3 via its LC3-interacting region (LIR) motif^107^. A N-terminal-truncated isoform of RETREG1/FAM134B, FAM134B-2, consists of 6 exons instead of 9 for the full-length isoform, regulates starvation-induced hepatic selective reticulophagy. FAM134B-2 regulates selective reticulophagy of secretory proteins such as APOC3 (apolipoprotein C3) and not bulk ER degradation through autophagy. RETREG1 is detected solely in the brain, spleen, and testis. Under starvation conditions FAM134B-2 expression is induced in peripheral tissues through the transcriptional activation of CEBPB/C/EBPβ(CCAAT enhancer binding protein beta)^108^. RTN3, belonging to the reticulon (RTN) protein family, is characterized by an anchoring to ER tubules^107^. The elucidation of distinct functions of RTN family members is a challenge considering the existence of a large number of RTN splice isoforms. Although all RTN splice variants contain a conserved C-terminal region, they exhibit major variations in their N-termini due to splicing rearrangements^109^. However, so far full-length RTN3 (RTN3L) was given a unique function in the regulation of tubular ER turnover via selective autophagy, whereas RTN3 cellular function remains unclear. Indeed, RTN3L, is the only RTN isoforms able to interact with MAP1LC3 upon starvation conditions via its six LIR domains, and to promote ER fragmentation and degradation through reticulophagy. To date, no pathologies have been associated with the lack or overexpression of RTN3L, whereas RTN3s aggregates have been found to induce neurite dystrophy and contribute to the cognitive failure in patients with AD^110^.

### Interplay between alternative splicing and autophagy response in human diseases

**Fig. 4:**
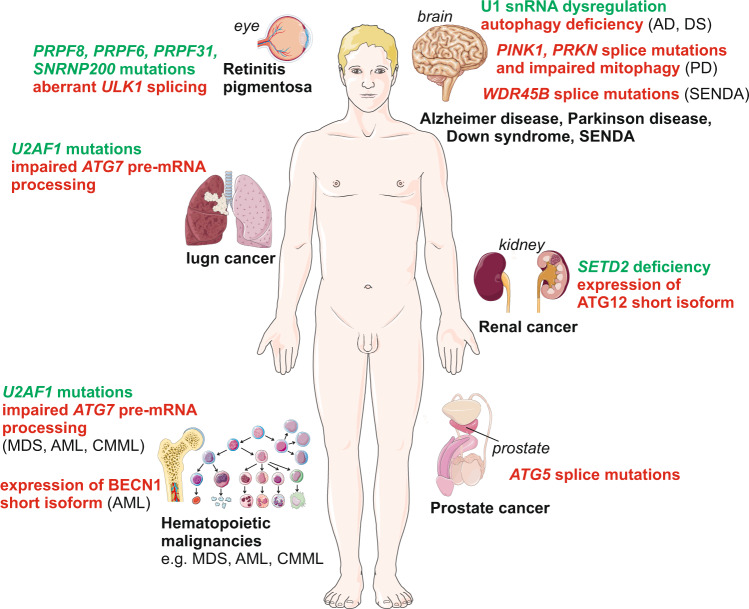
Human diseases associated with alternative-splicing dependent autophagy impairment. For details, see text body. In brief, U1 snRNP enrichment in familial Alzheimer disease (AD) and Down syndrome (DS) are associated with impaired autophagy. De novo WDR45/WIPI4 splice mutations and lowered autophagic flux are associated with static encephalopathy of childhood with neurodegeneration in adulthood (SENDA). Splicing mutations in *PINK1* and *PRKN*/*PARKIN* lead to mitophagy impairment and contribute to PD and various cancer. Aberrant alternative splicing of *ULK1* and impaired mitophagy associated with *PRPF8*, *PRPF6*, *PRPF31* and *SNRNP200* mutations contribute to retinitis-pigmentosa. *U2AF1*-mutation dependent ATG7 expression reduction, due to impaired pre-mRNA processing, in hematopoietic cells promotes autophagy impairment and oncogenic transformation, as observed in myelodysplastic syndrome (MDS), acute myeloid leukemia (AML), and chronic myelomonocytic leukemia (CMML) as well as lung adenocarcinoma. *BECN1* short isoform generated by alternative splicing acts as a negative modulator of autophagy in AML. Deficiency in *SETD2*, an epigenetic regulator of alternative splicing, promotes the expression of an ATG12 short isoform and alteration of autophagic flux in renal cancer. Splice-site mutations in *ATG5* lead to a dysregulation of autophagy in prostate cancer. Images from Servier Medical Art (smart.servier.com) licensed under a Creative Commons Attribution 3.0 Unported License (https://creativecommons.org/licenses/by/3.0/) were used to generate the depicted original illustration (smart.servier.com/terms-of-use/).

The contribution of dysfunctions in autophagy as well as the involvement of alteration in alternative mRNA splicing in human diseases have both been extensively reviewed separately^111,112^. Here, we discuss the importance of the interplay between autophagy and alternative splicing for human diseases (Fig. 4).

#### ATG5 splice mutation in prostate cancer

Valproic acid, a pan-histone deacetylase inhibitor, has been used in clinical trials to treat patients with castration-resistant prostate cancer^113^. Analysis of the autophagic process in a panel of prostate cancer cell lines, including androgen-sensitive prostate LNCaP cells as well as androgen-refractory PC-3 and DU145 cells, reveal clear differences in their autophagic response to valproic acid^114^. Unlike, PC-3 and LNCaP cells, DU145 prostate cancer cells do not show ATG5 expression and consequently are unable to form functional ATG12–ATG5 conjugation complexes, resulting in an impairment of LC3 lipidation. DU145 cells, due to an *ATG5* splice donor site mutation, express two alternative *ATG5* mRNAs that either lack exon 6 or exons 3 and 6, leading to premature termination of translation^92,114^. Recent in silico studies indicate that *ATG5* splice-site mutations that trigger exon skipping or introduction of a frameshift that might prevent ATG5 expression, are found in multiple tumor types, among them, cervical squamous cell carcinoma (exon 2 skipping), hepatocellular carcinoma (exon 3, 6 or 7 skipping) or uterine corpus endometrial carcinoma (exons 4 and 7 skipping), suggesting that alterations in *ATG5* mRNA splicing may not be limited to some prostate cancer cells^92^. However, it remains to be established whether these predicted splicing defects lead to loss of protein expression and/or loss of function for ATG5 as observed in prostate cancer cells.

#### Aberrant *ATG7* pre-mRNA maturation in hematopoietic malignancies

Prevalent *U2AF1* somatic mutations, including *U2AF1*^*S34F*^, occur in hematopoietic malignancies including myelodysplastic syndrome (MDS), AML, and chronic myelomonocytic leukemia (CMML), as well as in some solid tumors such as lung adenocarcinoma^115–117^. *U2AF1* mutations lead to impaired autophagy due to aberrant *ATG7* pre-mRNA processing, which was confirmed in MDS patient cells carrying the U2AF1 mutations S34F, S34Y or Q157P, which showed increased use of the distal cleavage and polyadenylation (CP) site in the *ATG7* transcript^118^. Bioinformatic analysis of the AML dataset contained in The Cancer Genome Atlas also revealed preferential usage of distal *ATG7* CP sites in patients carrying the mutation as compared to individuals with the wild-type genotype. Moreover, animal studies have shown that mice with hematopoietic stem cells lacking *Atg7* develop MDS-like syndrome, with severe myeloid cell proliferation, suggesting that ATG7 can act as regulator of hematopoietic stem cell maintenance^119^. U2AF1^S34F^-transformed Ba/F3 cells not only exhibit defects in autophagy, but also accumulate dysfunctional, non-respiring mitochondria and increased mitochondrial reactive oxygen species production, as well as an increase in spontaneous mutation frequency, as compared to control Ba/F3 cells. Knockdown of ATG7 expression in these cells, can mimic the effect of U2AF1^S34F^ mutant protein expression, suggesting that the decreased ATG7 expression, observed in U2AF1^S34F^-expressing Ba/F3 cells, contributes to the oncogenic transformation. MDS patients show abnormalities in various blood cells, including B cells, and granulocytes. However, it is important to note that whereas the usage of the distal CP site of *ATG7* is observed in U2AF1^S34F^ pro-B cells, it is not the case in *U2AF1*^*S34F*^ granulocytic cells^118^. Likewise, *U2AF1*^*S34F*^-expressing HCC78 cells do not exhibit alteration in the *ATG7* pre-mRNA maturation^25^, once more arguing for cell-type or lineage-specific effect of the mutation on both autophagy and oncogenic transformation. Reduced ATG7 expression has prognostic value in several cancers. For example, as compared to normal breast tissue, significantly lower expression of ATG7 is reported in triple-negative breast cancer and is considered an unfavorable clinicopathological factor for this breast cancer subtype^120^. However, whether the reduced ATG7 expression level in these cancer types is the result of alteration in the alternative-splicing machinery or another process remains to be explored; rescue of the defect by expression of wild-type ATG7 would be informative.

#### WDR45/WIPI4 splice mutations in SENDA neurodegenerative disorder

Static encephalopathy of childhood with neurodegeneration in adulthood (SENDA), is an X-linked dominant neurodegenerative disorder, characterized by deterioration and progressive accumulation of iron in the basal ganglia that begins early in childhood and worsens during early adulthood due to the development of parkinsonism and dementia. SENDA, results from de novo or autosomal recessive mutations. Exon sequencing of a family cohort described de novo WDR45 splice-site mutations in female patients, one nonsense mutation and two frameshift mutations. A c.439 + 1 G>T mutation generates a cryptic splice donor site by an insertion of 24 base pairs in intron 7, and a c.516G>C mutation produces a cryptic splice donor site due to an insertion of 22 base pairs located at the last nucleotide of exon 8. Analysis of the autophagy process in lymphoblastoid cells from the affected patients revealed inefficient autophagosome formation and lowered autophagy flux^75^. The analysis of central nervous system-specific *Wdr45* knockout mice revealed autophagy defects as well as the development of axonal swelling and behavioral abnormalities, including motor deficits and learning and memory impairment^121^. Therefore, WDR45 should be considered as guarding central nervous system homeostasis, because *WDR45* splice mutations can trigger neurodegenerative disorders.

#### Dysregulation of ATG12 splice isoforms ratio in clear cell renal cell carcinoma

As previously mentioned, in ccRCC cells, *SETD2* deficiency is associated with the appearance of free ATG12, as well as the expression of additional ATG12-containing complexes, along with the recognized ATG12–ATG5 conjugate, and an overall increase in total ATG12 protein expression levels. In accord with these inverse observations, in ccRCC patients whereas high *SETD2* gene expression levels are associated with a favorable prognosis, high *ATG12* gene expression levels are associated with an unfavorable prognosis^53^. SETD2 rescue in deficient ccRCC cells not only affects the presence of the additional ATG12-containing complexes as well as free ATG12L isoforms, but as also the autophagic flux. Furthermore, the SETD2-mediated manipulation of ATG12 isoforms expression ratio in these cancer cells has an impact on their migration capability. Collectively, these findings bring further argument for considering the *SETD2* gene status of ccRCC tumors, when therapeutic interventions, such as targeting the autophagic process, are considered to combat these kidney cancers. Regardless of the findings that revealed *SETD2*-mediated regulation of autophagy via ATG12, it would of interest to have additional research focused on the potential regulation of other autophagy-related gene isoforms as well as potential regulators by SETD2 not only in ccRCC, but also in other types of cancer or neurodegenerative diseases. It is worth noting that *SETD2* mutations were first reported in patients with the Sotos syndrome, a genetic disorder characterized by overgrowth and a non-progressive neurological disability^122^. Beyond the reported inactivation of *SETD2* in ccRCC cancer, whole-exome sequencing studies also revealed somatic *SETD2* mutations in various types of cancer, albeit mostly at low frequencies^123^. Hence, SETD2 inactivation could play a role in the development of other tumors as well as the Sotos syndrome disorder. However, whether these genetic alterations affect the autophagic process remains to be explored.

#### BECN1 splice isoforms in leukemia

The *BECN1* gene is essential for early embryonic development and its perturbation is linked to the onset of several human diseases including cancer, and cardiovascular and neurodegenerative diseases, as well as the response to pathogen infection^124^. In fact, *Becn1-*deficient mice die before birth, whereas *Becn1* haploinsufficient mice, which exhibit reduced autophagy, suffer from a high incidence of spontaneous tumors^125,126^. In human, monoallelic deletion of *BECN1* is observed in 30 to 75% of cases of different types of cancers, including lymphoma, hepatocellular, lung, ovarian, gastric and breast cancers, and the low expression of BECN1 is associated with worse overall survival and/or cancer progression-free survival of patients^127,128^. However, it is important to note that the proximity of *BECN1* and *BRCA1* loci on chromosome 17q21 has made the determination of the impact of *BECN1* gene alteration equivocal, as effects attributed to *BECN1* gene alteration could be due to *BRCA1* instead^129,130^. A transcript variant of the *BECN1* gene carrying a deletion of exon 11, BECN1S, which encodes a C-terminal truncated BECN1 splice isoform, is reported in human B-cell acute lymphoblastic leukemia cells. This BECN1 isoform displays reduced activity in the induction of autophagy in response to starvation. Therefore, the BECN1S isoform is suggested to act as a negative regulator of autophagy competing with the canonical BECN1 isoform that can contribute to the development of human B-cell acute lymphoblastic leukemia^68^.

#### Aberrant ULK1 alternative splicing in retinitis pigmentosa

As described above, PRPF8 is a regulator of the spliceosome core machinery, and *PRPF8* knockdown is associated with aberrant *ULK1* mRNA splicing and deficient mitophagy^22^. Mutations in *PRPF8* contribute to pathogenesis of autosomal dominant retinitis pigmentosa, a hereditary degenerative eye disease with a progressive loss of photoreceptor cells^131^. The retinal cell environment is characterized by low oxygen levels due to the high oxygen demand of the photoreceptors, and hypoxia is reported to induce mitophagy. In retinal cells, silencing of *PRPF8* gene expression, through the dysfunctional regulation of *ULK1* alternative splicing, leads to impairment of mitophagosome formation and mitochondrial clearance. Remarkably, whereas expression of wild-type PRPF8 protein can rescue the hypoxic mitophagy defect observed in *PRPF8* knockdown cells, expression of PRPF8^R2310K^, an autosomal dominant retinitis-pigmentosa associated mutant, is ineffective in doing so^22^. Interestingly, this degenerative disease is characterized by mutations in other core spliceosome components including *PRPF6*, *PRPF31*, and *SNRNP200* (small nuclear ribonucleoprotein U5 subunit 200)^131–133^ whose downregulation is also connected to *ULK1* mRNA mis-splicing and mitophagy defects^22^.

#### PINK1 and PRKN splicing mutations in parkinsonism and various cancers

Approximately 10% of patients with Parkinson disease (PD) present with a recessive early-onset, defined by a diagnosis before 50 years of age. Genetic factors, including mutations in the *PRKN* and *PINK1* genes contribute to early-onset PD. Homozygous or compound heterozygous mutations of the *PRKN* gene are found in more than half of the cases of autosomal recessive forms of PD. Heterozygous deletion of exon 1, 2, 4, and/or 5 in *PRKN* is a common feature of patients for early-onset PD^134,135^. *PRKN* deficiency has also been linked to other human pathologies, including cancer^136,137^. Evidence suggests that PRKN can function as a tumor suppressor. Mutations in *PRKN* abrogate the growth-suppressive effects of wild-type PRKN in different human cancer cell lines^137,138^. The autosomal recessive pattern of inheritance of *PINK1* mutations suggests that PINK1 is neuroprotective and therefore loss of PINK1 function causes early-onset parkinsonism. Whereas exon rearrangements are frequently encountered in the *PRKN* gene, most *PINK1* mutations are represented by single nucleotide changes that generate nonsense mutations. However, whole-exome sequencing and Sanger sequencing identified several splicing mutations in exon 7 of the *PINK1* gene, which affects pre-mRNA splicing^139,140^. These single nucleotide mutations at the 5′ splice-site result in skipping of the mutation-harboring exon 7 likely resulting from an inefficient interaction of the U1 snRNP at the donor site^141^. These PD-associated mutations in *PINK1*, appears to significantly reduce its kinase activity^142^.

### Targeting alternative splicing to affect autophagy as a therapeutic strategy

There is undoubtedly convincing evidence that establishes a substantial association between the alternative-splicing machinery and the regulation of autophagy, with the existence of splice isoforms for the majority of components of the autophagy core machinery. Remarkably, most of these protein isoforms, encoded by a single autophagy-related gene, are shown to exert distinct and sometimes even opposite roles in the regulation of autophagy. They are also found to have an impact on the process at all steps, from the initiation of phagophore formation (e.g., BECN1, ATG14 isoforms), and the expansion of the phagophore (e.g., ATG10, ATG12, ATG16L1 isoforms) to the completing of the flux with the fusion of autophagosomes with lysosomes (e.g., ATG10, ATG14, LAMP2 isoforms). Furthermore, autophagy-regulated genes splice mutations (e.g., *ATG5*, *PINK1, PRKN, WDR45/WIPI4*), and alteration in splicing factors associated with an impairment of the autophagy process including the aberrant maturation of *ATG* pre-mRNA (e.g., *ULK1, ATG7, ATG12*), have both been linked to various human diseases ranging from neurodevelopment to neurodegenerative disorders, including neoplastic malignancies.

The dysregulation of the mRNA splicing machinery in human diseases provides therapeutic opportunities (Table 1). In fact, **small-molecule modulators of mRNA splicing** that target RBPs and act as inhibitors or activators are used as clinical drugs. In agreement with the reported interplay between the alternative-splicing machinery and the autophagy process, intervention aiming at the regulation of splicing factors also have an impact on autophagy among other cellular pathways. Targeting the deregulated spliceosome core machinery in various cancer cell types, via the genetic inhibition of SNRPE, or SNRPD1, triggers MTOR blockade, which is well known to cause autophagy induction^46^. Spliceostatin A1, pladienolide B and herboxidiene compounds used in the clinic all target the stage where U2 snRNP joins the branch point sequence and robustly inhibits alternative splicing. These drugs bind to and inhibit the same U2 snRNP component, SF3B1, and thereby exert potent anti-tumoral effects (reviewed in ref. ^143^). There are currently no reports that shows that these drugs are involved in the regulation of autophagy by directly targeting alternative splicing. However, these compounds all target SF3B1, whose mutation in MDS patients is associated with differential expression of autophagy-related genes including *ULK1*, *ATG16L2*, and *ATG9A*^144^, which brings further support to the link between these two cellular regulatory pathways. Moreover, a low dose of pladienolide B reduces the expression of genes involved in selective autophagy in HEK293 cells^145^. Bromodomain (BET) proteins, histone modification epigenetic sensors, contribute to the regulation of alternative splicing, and BET inhibitors, including the JQ1 compound, are extensively used in cancer treatment^146,147^. It is worth noting that BET inhibitors are also potent inducers of autophagy, and induction of autophagy is in turn reported to contribute to the observed acquired resistance of cancer cells to BET inhibitors^147,148^. Therefore, BET inhibitors are recommended to be used in combination therapy. Quercetin, a polyphenolic HNRNPA1 inhibitor compound, enhances the anti-tumoral effects of BET inhibitors in a pancreatic cancer animal model^149^. However, it is important to note that quercetin as a single treatment is reported to trigger protective autophagy in various cancer cells that could potentially lead to cancer cell resistance, as reported for the BET inhibitors^150,151^. Given the autophagy-dependent acquisition of cancer resistance upon BET inhibitor treatment, the effect of autophagy inhibition has to be considered in that context. In fact, inhibition of autophagy by pharmacological inhibitors or siRNA-mediated knockdown of *BECN1* specifically enhance JQ1-induced apoptosis in BET inhibitor-resistant AML cells^152^. Hence, inhibition of autophagy could be an effective therapeutic strategy for combating resistance to BET inhibitors in cancer. Nevertheless, numerous small-molecule modulators of RNA splicing affect autophagy and appear to provide a successful treatment with a promising potential to be further translated to the clinic.

**Table 1:** Drugs used to target alternative splicing and impacts on autophagy.

Drugs	Function and target	Diseases	Ref.
*Small-molecule splicing modulators*			
Spliceostatin A1	SF3B1 inhibitor	–	^143^
Pladienolide B	SF3B1 inhibitor	Myelodysplastic syndrome	^143–145^
Herboxidine	SF3B1 inhibitor	–	^143^
BET inhibitors	Regulation of alternative splicing and autophagy inducer	–	^146–148^
Quercetin	HNRNPA1 inhibitor	Cancer	^149–151^
*Small-molecule autophagy modulators*			
Rapalogs	MTOR inhibitors, autophagy inducers and TRIB3-mediated alternative splicing	Cancer; neurodegenerative diseases	^153^
Carbamazepine (CBZ)	Autophagy inducer	Epilepsy; bipolar disorders; metaphyseal chondrodysplasia, Schmid type	^154^
Chloroquine (CQ)	Autophagy inhibitor via autophagosome-lysosome fusion; HNRNPK inhibitor	Myotonic dystrophy type 1	^155–157^
*Antisense morpholino oligonucleotides*			
Specific-target AMOs candidate	pre-mRNAs splicing manipulation. Targets splicing motifs to: reinforce exon selection, excise exons that contain nonsense mutations or those that flank frameshifting rearrangements.	Duchenne muscular dystrophy	^158^
*Small-molecule targeting RNA/RNA-protein complexes*			
Risdiplam	The drug promotes the inclusion of exon 7 in *SMN2* and thereby the production of SMN2 protein.	Spinal muscular atrophy	^163–165^

However, it should be noted that the reverse is also true: **small-molecule modulators of autophagy** have an impact on the splicing machinery. Rapalogs, rapamycin derivatives, small-molecule MTOR inhibitors and autophagy inducers, have become standard-of-care for patients with breast, kidney, and neuroendocrine cancers. However, resistance to therapy occurs usually after several months of treatment. Rapalog-mediated repression of TRIB3 (tribbles pseudokinase 3), which interacts with the pre-mRNA splicing machinery, induces the dysregulation of spliceosome function that could contribute to the observed therapeutic resistance^153^. Carbamazepine, another autophagy inducer, is approved for the treatment of epilepsy and bipolar disorders. Treatment of patients with dwarfism metaphyseal chondrodysplasia Schmid type (MCDS), characterized by the accumulation of misfolded collagen X on the ER that affects cell differentiation and bone growth, with Carbamazepine promotes an increase of proteolysis and degradation of misfolded COL10A1/collagen X. An animal model of MCDS suggest that carbamazepine treatment has an impact on the alternative-splicing pre-mRNA of the unfolded protein response effector *XBP1* (X-box binding protein 1)^154^. Likewise, small-molecule inhibitors of autophagy are reported to exert effects on the alternative-splicing machinery. For example, quinolone derivatives can bind and inhibit the HNRNPK splicing factor^155^. Chloroquine (CQ), an inhibitor of autophagosome-lysosome fusion, is proposed for the treatment of myotonic dystrophy type 1 (DM1), a debilitating neuromuscular disease caused by the expansion of a CUG repeat in the 3′ UTR of the *DMPK* (DM1 protein kinase) pre-mRNA. The mutant extended pre-mRNA forms insoluble structures capable of sequestering RBPs of the MBNL (muscleblind like splicing regulator) family; their limited availability in turn leads to altered alternative splicing and ultimately the disease phenotype. Treatment with CQ upregulates MBNL1 and MBNL2 protein expression and thereby improves DM1 phenotypes in patient-derived myoblasts and animal models of the disease^156^. Furthermore, an early study even revealed that CQ can significantly enhance so-called in vitro splice correction activity^157^. As a note of caution, the idea of targeting autophagy to combat cancer is complicated and requires further understanding of the disease per se. For instance, the dual role of autophagy at different stages of tumorigenesis, requires the action of several treatments that either activate or inhibit autophagy but also target other cellular pathways, especially in multifactorial diseases such as cancer.

It should be noted that the protein isoforms for autophagy core components generated by alternative splicing, as well as the aberrant splice variants observed in the context of diseases, exert their effects at specific steps of the autophagy process, and the targeting of isoforms working in the initiation, or the completion of autophagy will generate entirely distinct cellular phenotypes. Therefore, therapeutic interventions aiming at the manipulation of one specific autophagy-related protein isoform potentially acting at a deficient autophagy step of interest in one disease, or the re-expression of a wild-type isoform from a splice mutation variant could be of therapeutic interest. In fact, such an approach has already been undertaken through **antisense oligonucleotide (ASO)-mediated splice-switching** and shown to be successful. ASOs have been used to manipulate the splicing of pre-mRNAs through targeting splicing motifs to reinforce exon selection, or excise exons that contain nonsense mutations or those that flank frameshifting rearrangements, to produce wild-type transcripts as a potential treatment for several inherited disorders^158^. The implementation in clinics of two antisense RNA therapeutics, Exondys 51 to treat Duchenne muscular dystrophy and Spinraza as a treatment for spinal muscular atrophy (SMA) confirm the therapeutic potential of ASOs^159^. For example, in the case of the Duchenne muscular dystrophy, ASO-mediated manipulation of pre-messenger RNA splicing bypass the Duchenne-causing mutations in *DMD* (dystrophin) and restore functional expression of the DMD protein. Would such intervention be applicable for the regulation of specific autophagy-related splice isoforms? As described above, ATG10S (short) and ATG10 (canonical long) are two splice isoforms which exert distinct functions in the autophagic process in response to HCV infection. As a proof of concept, a morpholino oligo can switch the expression of ATG10 to ATG10S by promoting the removal of exon 4 in the *ATG10* pre-mRNA and affect both the autophagy flux and the replication of the virus^81^. The potential benefit of ASOs for the manipulation of alternative-splicing dependent autophagic functions is limitless, and includes the restoration of ATG5, ATG7, and ATG16L1 wild-type expression in cancer cells, the abrogation of ATG12S or BECN1S expression, the selective expression of PINK1FL to promote mitophagy, and the selective control of ATG14S and ATG14L expression to potentially regulate autophagy induction or completion.

Another possible strategy to correct aberrant alternative splicing of autophagy-related mRNAs, could rely on the use of **small-molecule drugs that act by directly targeting RNA or RNA-protein complexes**^160,161^. We hypothesize that future development may lead to the generation of biologically active small molecules that specifically target mRNA for an autophagy-related gene, affect its splicing and in turn affect the function(s) carried out by one isoform encoded by that gene. In fact, such a small-molecule drug already exists to correct the splicing of *SMN2* (survival of motor neuron 2, centromeric) in the context of SMA. SMA is caused by the loss or mutation of both copies of the *SMN1* (survival of motor neuron 1, telomeric) gene^162^. The related *SMN2* gene is retained, but due to alternative splicing of exon 7, produces insufficient levels of the SMN protein. Risdiplam promotes the inclusion of exon 7 and increases production of SMN2 protein in human cells^163,164^. This drug stabilizes the interaction between the 5′ splice site of exon 7 and the U1 snRNP of the spliceosome^165^. Risdiplam, sold under the brand name Evrysdi, and acting as a *SMN2*-directed RNA splicing modifier is used as an FDA-approved oral medication to treat SMA.

Overall, the discovery that protein isoforms are produced by the majority of genes encoding components of the core autophagy machinery and that these alternative spliced isoforms of autophagy-related genes differentially affect the autophagy machinery bring an additional level of complexity in the understanding of the regulation of this biological process. At the same time, these discoveries shed light on the potential therapeutic benefit of targeting the interplay between alternative-splicing machinery and autophagy core components. One can be persuaded that further advances allowing us to selectively regulate the expression and functions of individual protein isoforms will be the pavement for the development of novel therapeutic avenues.

## Supplementary information

Supplementary Information
