# Supplementary material for: Autophagy regulation by RNA alternative splicing and implications in human diseases
Source: Nat Commun. 2022 May 18;13:2735. doi: 10.1038/s41467-022-30433-1 (PMC9117662; doi:10.1038/s41467-022-30433-1)
Supplement: Supplementary file 1 — Supplementary Information [file 41467_2022_30433_MOESM1_ESM.pdf]

Supplementary Note 1 | List of abbreviations.

3'SS, 3' splice site; **5'SS**, 5' splice site; **AD**, Alzheimer disease; **AMBRA1**, autophagy and beclin 1 regulator 1; **AML**, acute myeloid leukemia; **AMPK**, AMP-activated protein kinase; **APOC3**, apolipoprotein C3; **APP**, amyloid beta precursor protein; **ASO**, antisense oligonucleotide; **ATAT1**, alpha tubulin acetryltransferase 1; **ATG**, autophagy related; **ATG16L1**, autophagy related 16 like 1; **ATL3**, Atlastin GTPase 3; **BCL2L1**, BCL2 like 1; **CCD**, coiled-coil domain; **BNIP3**, BCL2 interacting protein 3; **BNIP3L**, BCL2 interacting protein 3 like; **CCPG1**, cell cycle progression 1; **ccRCC**, clear cell renal cell carcinoma; **CEBPB**, CCAAT enhancer binding protein beta; **CMA**, chaperone-mediated autophagy; **CMML**, chronic myelomonocytic leukemia; **CP**, cleavage and polyadenylation; **CQ**, chloroquine; **DS**, Down syndrome; **ER**, endoplasmic reticulum; **ESE**, exonic splicing enhancer; **ESS**, exonic splicing silencers; **FOXO1**, forkhead box O1; **GABARAP**, GABA type A receptor-associated protein; **H3K36me3**, trimethylation of lysine 36 on histone H3; **H3K4me3**, trimethylation of lysine 4 on histone H3; **HCV**, hepatitis C virus; **HDAC6**, histone deacetylase 6; **hnRNP**, heterogeneous ribonucleoprotein; **HSPA8**, heat shock protein family A (Hsp70) member 8; **HSP8A**, heat shock protein family A (Hsp70) member 8; **IFNL2**, interferon lambda 2; **LAP**, LC3-associated phagocytosis; **LAMP2,** lysosomal associated membrane protein 2; **LIR**, LC3-interacting region; **MAP1LC3/LC3**, microtubule associated protein 1 light chain 3; **MBNL**, muscleblind like splicing regulator; **MDS**, myelodysplastic syndrome; **MFN2**, mitofusin 2; **MORF4L1**, mortality factor 4 like 1; **MTOR**, mechanistic target of rapamycin kinase; **PD**, Parkinson disease; **PE**, phosphatidylethanolamine; **PINK1,** PTEN induced kinase 1; **PIK3C3**, phosphatidylinositol 3-kinase catalytic subunit type 3; **PIK3R4**, phosphoinositide-3-kinase regulatory subunit 4; **Poly(A)**, polyadenylation; **PRKN**, parkin RBR E3 ubiquitin protein ligase; **PRPF8**, pre-mRNA processing factor 8; **PSEN1**, presenilin 1; **PTBP1**, polypyrimidine tract binding protein 1; **PtdIns3K**, class III phosphatidylinositol 3-kinase; **PtdIns3P**, phosphatidylinositol-3-phosphate; **PTEN**, phosphatase and tensin homolog; **RAB33B**, RAB33B, member RAS oncogene family; **RBCC1**, RB1-inducible coiled-coil protein 1; **RBFOX2**, RNA binding fox-1 homolog 2; **RBP**, RNA-binding protein; **RETREG1**, reticulophagy regulator 1; **RELA**, RELA proto-oncogene, NF-kB subunit**RTN3,** reticulon 3; **RUBCN**, rubicon autophagy regulator; **SENDA**, static encephalopathy of childhood with neurodegeneration in adulthood; **SETD2**, SET domain containing 2, histone lysine methyltransferase; **SF1**, splicing factor 1; **SMAC**, single-membrane Atg8-family protein conjugation; **SNAP29**, synaptosome associated protein 29; **SNARE**, soluble N-ethylmaleimide-sensitive factor attachment protein receptor; **SEC62**, SEC62 homolog, preprotein translocation factor;**SMA**, spinal muscular atrophy; **SMN1**, survival of motor neuron 1, telomeric; **SMN2**, survival of motor neuron 2, centromeric; **snRNA**, small nuclear RNA; **snRNP**, small nuclear ribonucleoprotein; **SNRPD1**, small nuclear ribonucleoprotein D1 polypeptide; **SNRPE,** small nuclear ribonucleoprotein polypeptide E; **SNRNP200**, Small Nuclear Ribonucleoprotein U5 Subunit

200; **SQSTM1/p62**, sequestosome 1; **SRSF**, serine and arginine rich splicing factor; **STX17**, syntaxin 17; **TEX264**, testis expressed 264, ER-phagy receptor; **TRIB3**, Tribbles Pseudokinase 3; **Ub**, ubiquitin; **Ubl**, ubiquitin like; **ULK1**, unc-51 like autophagy activating kinase 1; **U2AF**, U2 small nuclear RNA auxiliary factor; **VAMP8**, vesicle associated membrane protein 8; **WDFY3,** WD Repeat And FYVE Domain Containing 3; **WDR45**, WD repeat domain 45; **WIPI**, WD repeat domain, phosphoinositide-interacting; **XBP1**, X-box binding protein 1; **ZFYVE1**, zinc finger FYVE-type containing 1.

Supplementary Table 1 | Alteration in the alternative splicing of autophagy-related genes and impact on diseases

Autophagy-related gene	Variants	RNA splicing event(s)	Cellular function/ phenotype	Disease-associated variants	References
Initiation					
ULK1	*ULK1(1) – canonical variant*	28 exons	Autophagy induction and autophagosome biogenesis	Cancer; neurodegenerative diseases; neurodevelopment disorders; Crohn disease	[1]
	ULK1(2) – generated by splicing mutation	Skipping exon 22	Mitophagosome formation. Hypoxia-induced mitophagy	Autosomal dominant retinitis pigmentosa	[2]
	ULK1(3) – generated by splicing mutation	Skipping exon 22-23	Mitophagosome formation. Hypoxia-induced mitophagy	Autosomal dominant retinitis pigmentosa	[2]
Nucleation					
BECN1	*BECN1 Long – canonical variant*	12 exons	Autophagy induction	-	[1,3-6]
	BECN1S – generated by splicing mutation	Skipping exon 11; truncated C-terminus	Low binding affinity with class III PtdIns3K; Fail to activate autophagic response to starvation	Prostate cancer; Acute myeloid leukemia; acute lymphoblastic leukemia; B-cell acute lymphoblastic leukemia cells	[1,7,8]
	BECN1s – generated by splicing mutation	Skipping exons 10-11	Unable to initiate autophagy, but supports mitophagy	Acute myeloid leukemia	[1,7,8]

ATG14	*ATG14 Long – canonical variant*	9	Phagophore formation and autophagosome-lysosome fusion	Vici syndrome; Danon disease; cancer; neurodegenerative diseases	[9]
	ATG14 Short	Skipping exons 1-3	Inhibit autophagosome-lysosome fusion	-	[9]
WIPI1	*WIPI1α – canonical variant*	13 exons	Recruitment of ATG12–ATG5-ATG16L1 complex to nascent autophagosomes	-	[10,11]
	WIPI1β	-	Localizes to endosomal and golgi membranes	-	[12]
WIPI2	*WIPI2A – canonical variant*	13 exons	Involved in the formation of autophagosomal structures: mediates ER-phagophore contacts: recruits the ATG12–ATG5-ATG16L1 complex.	-	[10,11]
	WIPI2B	Skipping exon 12; 3' truncation end	Interacts with ATG16L1; required for innate immune response to *Salmonella* Typhimurium infection	-	[10,11,13]

	WIPI2C	-	-	-	[10,11]
	WIPI2D	Skipping exon 2; 3' truncation end	Promotes LC3 lipidation	-	[14]
	WIPI2E	-	-	-	[11]
WIPI4	*WIPI4α –canonical variant*	11 exons	PtdIns3P binding effector, autophagosome biogenesis and size control	Neurological disorders	[15,16]
	WIPI4β	Skipping exon 3-4	-	Static encephalopathy of childhood with neurodegeneration in adulthood	[15]
	WIPI4Δ	Early 3' end termination exon 11	-	Static encephalopathy of childhood with neurodegeneration in adulthood	[15]
Phagophore expansion					
ATG7	*ATG7 long – canonical variant*	19 exons	LC3/GABARAP lipidation	Neurodegeneration; hepatic diseases; cardiovascular diseases	[17-19]

	ATG7 short	Skipping exon 17	Inhibit autophagy. Unable to bind to LC3	Myelodysplastic syndrome; Acute myeloid leukemia; chronic myelomonocytic leukemia	[20,21]
	ATG7 (3)	Skipping exons 17-18	-	-	[20,21]
MAP1LC3A	*MAP1LC3A – canonical variant*	4 exons	Autophagosome biogenesis; adaptor selective cargo for autophagy	Alzheimer disease; Machado-joseph disease; cancer	[22]
	MAP1LC3A-b	Additional exon	-	-	[22]
MAP1LC3B	*MAP1LC3B - canonical variant*	4 exons	Autophagosome biogenesis; adaptor selective cargo for autophagy	Hermansky-pudlak syndrome; Parkinson disease 1, autosomal dominant; cancer	[22,23]
	MAP1LC3B-a	NAGNAG splice sites of intron 3	Inhibits interaction and binding with ATG4B	-	[22,23]
GABARAP	*GABARAP – canonical variant*	4 exons	Autophagosome biogenesis; receptor of selective cargo for autophagy	Stiff-Person syndrome; Machado-joseph disease; Parkinson disease; colorectal carcinoma	[24]

	GABARAP-a	Different 3' end points	Inhibits interaction and binding with ATG4	-	[24]
GABARAPL1	*GABARAPL1*	4 exons	Autophagosome biogenesis; receptor of selective cargo for autophagy	Vici syndrome; Alzheimer disease; cancer	[24]
	GABARAPL1-a	Different 3' end points	Inhibits interaction and binding with ATG4	-	[24]
Autophagosome completion					
ATG10	*ATG10 long (ATG10L) – canonical variant*	8 exons	Formation of the ATG12–ATG5 complex	Cancer; infection; inflammation	[25-27]
	ATG10 short (ATG10S)	Early termination 3'; Skipping exon 4	Autophagy induction in HCV infection; transcription factor	Hepatitis C virus infection	[25,28,29]
ATG12	*ATG12 long (ATG12L)*	4 exons	Autophagosome biogenesis; proviral factor; required for HCV translation.	Clear cell renal cell carcinoma; neurodegenerative diseases	[30]
	ATG12 short (ATG12S)	Alternative exon 2	Decrease autophagy flux	SETD2-deficient clear cell renal cell carcinoma	[30]

ATG5	*ATG5 long*	11 exons	Autophagosome formation, LC3 lipidation and mitochondria quality.	Neurodegenerative diseases; cardiovascular diseases; pathogen infection; immune diseases	[17]
	ATG5 Short	Skipping exon 6 or exons 3 and 6	Unable to form ATG12–ATG5 complex	Prostate DU145 cancer cells	[31,32]
	ATG5 short – splice site mutation	Skipping exon 2	Unable to form ATG12–ATG5 complex	Cervical squamous cell carcinoma	[33]
	ATG5 short (1) – splice site mutation	Skipping exon 3, 6 or 7	Unable to form ATG12–ATG5 complex	Hepatocellular carcinoma	[33]
	ATG5 short (2) – splice site mutation	Skipping exon 4 and 7	Unable to form ATG12–ATG5 complex	Uterine corpus endometrial carcinoma	[33]
ATG16L1	*ATG16L1α*	Skipping exons 8-9	Unable to lipidate LC3	Crohn disease; gastric cancer; brain metastasis in lung cancer	[34-38]
	ATG16L1β – canonical variant	Skipping exon 9	ATG12–ATG5 conjugation and LC3 lipidation	Crohn disease; gastric cancer; brain metastasis in lung cancer	[36,39,17,40]
	ATG16L1γ	All 20 exons	-	-	[36]

ATG16L2	*ATG16L2 Long - canonical variant*	18 exons	May play a role in regulating epithelial homeostasis in an ATG16L1-dependent manner	Crohn disease; systemic lupus erythematosus	[17,33,40-42]
	ATG16L2 short	Skipping exon 8	-	-	[42]
**Autophagosome-lysosome function**					
LAMP2	*LAMP2A – canonical variant*	9 exons	Involved in CMA	Danon disease; hypertrophic cardiomyopathy; neurological diseases; cancer	[17,43]
	LAMP2B	9 exons	Involved in macroautophagy. Interacts with ATG14 and VAMP8 to promote fusion	Danon disease; hypertrophic cardiomyopathy; renal cell carcinoma	[17,43,44]
	LAMP2C	9 exons	Interact with RBPs and histone 1. Involved in RNautophagy and DNautophagy; negative regulator of CMA	Danon disease; hypertrophic cardiomyopathy	[17,43,45,46]
Mitophagy					
PINK1	*PINK1FL – canonical variant*	8 exons	Located at the mitochondria, involved in mitochondria clearance	Parkinson disease; Alzheimer disease	[47,48]
	PINK1-cyto	-	Located at the cytosol. Sequester ubiquitinated proteins into aggresomes	Parkinson disease; Alzheimer disease	[47,48]

	PINK1A – splice site mutation in exon 7	Skipping exon 7	-	-	[49,50]
PRKN	*PRKN H1 – Canonical variant*	12 exons	Mitophagy	Parkinson disease; Alzheimer disease; cancer	[51,52]
	PRKN H2	-	-	-	[52]
	PRKN H20	-	-	-	[52]
Reticulophagy					
RETREG1/ FAM134B	*FAM134B – canonical variant*	*9 exons*	Located in ER sheets, mediates ER remodeling by direct binding with LC3. Located in brain, spleen and testis.	Neurological diseases; viral replication; inflammation; cancer	[53,54]
	RETREG1/FAM134B-2	*6 exons*	Regulate selective reticulophagy of secretory proteins. Located in peripheral tissues.	-	[54]
RTN3	*RTN3L – canonical variant*	*9 exons*	Interact with MAP1LC3. Promote ER fragmentation and degradation.	Alzheimer disease; astrocytoma	[55,56]
	RTN3s	-	Unable to facilitate ER degradation. Induces neurite dystrophy	Alzheimer disease	[55]

Supplementary References

1 Wirawan, E. *et al.* Beclin1: a role in membrane dynamics and beyond. *Autophagy* **8**, 6-17, doi:10.4161/auto.8.1.16645 (2012).

2 Xu, G. *et al.* Autosomal dominant retinitis pigmentosa-associated gene PRPF8 is essential for hypoxia-induced mitophagy through regulating ULK1 mRNA splicing. *Autophagy* **14**, 1818-1830, doi:10.1080/15548627.2018.1501251 (2018).

3 Yue, Z., Jin, S., Yang, C., Levine, A. J. & Heintz, N. Beclin 1, an autophagy gene essential for early embryonic development, is a haploinsufficient tumor suppressor. *Proc Natl Acad Sci U S A* **100**, 15077-15082, doi:10.1073/pnas.2436255100 (2003).

4 Qu, X. *et al.* Promotion of tumorigenesis by heterozygous disruption of the beclin 1 autophagy gene. *J Clin Invest* **112**, 1809-1820, doi:10.1172/jci20039 (2003).

5 Zheng, T., Li, D., He, Z., Feng, S. & Zhao, S. Prognostic and clinicopathological significance of Beclin-1 in non-small-cell lung cancer: a meta-analysis. *Onco Targets Ther* **11**, 4167-4175, doi:10.2147/ott.s164987 (2018).

6 Nicotra, G. *et al.* Autophagy-active beclin-1 correlates with favourable clinical outcome in non-Hodgkin lymphomas. *Mod Pathol* **23**, 937-950, doi:10.1038/modpathol.2010.80 (2010).

7 Niu, Y. N. *et al.* Alternative messenger RNA splicing of autophagic gene Beclin 1 in human B-cell acute lymphoblastic leukemia cells. *Asian Pacific journal of cancer prevention : APJCP* **15**, 2153-2158 (2014).

8 Cheng, B. *et al.* BECN1s, a short splice variant of BECN1, functions in mitophagy. *Autophagy* **11**, 2048-2056, doi:10.1080/15548627.2015.1100785 [doi] (2015).

9 Diao, J. *et al.* ATG14 promotes membrane tethering and fusion of autophagosomes to endolysosomes. *Nature* **520**, 563-566, doi:10.1038/nature14147 (2015).

10 Dooley, H. C. *et al.* WIPI2 links LC3 conjugation with PI3P, autophagosome formation, and pathogen clearance by recruiting Atg12-5-16L1. *Mol Cell* **55**, 238-252, doi:10.1016/j.molcel.2014.05.021 (2014).

11 Mauthe, M. *et al.* Resveratrol-mediated autophagy requires WIPI-1-regulated LC3 lipidation in the absence of induced phagophore formation. *Autophagy* **7**, 1448-1461, doi:10.4161/auto.7.12.17802 (2011).

12 Jeffries, T. R., Dove, S. K., Michell, R. H. & Parker, P. J. PtdIns-specific MPR Pathway Association of a Novel WD40 Repeat Protein, WIPI49. *Molecular Biology of the Cell* **15**, 2652-2663, doi:10.1091/mbc.e03-10-0732 (2004).

13 Stavoe, A. K., Gopal, P. P., Gubas, A., Tooze, S. A. & Holzbaur, E. L. Expression of WIPI2B counteracts age-related decline in autophagosome biogenesis in neurons. *Elife* **8**, doi:10.7554/eLife.44219 (2019).

14 Fracchiolla, D., Chang, C., Hurley, J. H. & Martens, S. A PI3K-WIPI2 positive feedback loop allosterically activates LC3 lipidation in autophagy. *Journal of Cell Biology* **219**, doi:10.1083/jcb.201912098 (2020).

15 Zhao, Y. G. *et al.* The autophagy gene Wdr45/Wipi4 regulates learning and memory function and axonal homeostasis. *Autophagy* **11**, 881-890, doi:10.1080/15548627.2015.1047127 (2015).

16 Saitsu, H. *et al.* De novo mutations in the autophagy gene WDR45 cause static encephalopathy of childhood with neurodegeneration in adulthood. *Nature genetics* **45**, 445-449, 449e441, doi:10.1038/ng.2562 (2013).

17 Levine, B. & Kroemer, G. Autophagy in the pathogenesis of disease. *Cell* **132**, 27-42 (2008).

18 Mortensen, M. *et al.* The autophagy protein Atg7 is essential for hematopoietic stem cell maintenance. *J Exp Med* **208**, 455-467, doi:10.1084/jem.20101145 (2011).
19 Ilagan, J. O. *et al.* U2AF1 mutations alter splice site recognition in hematological malignancies. *Genome Res* **25**, 14-26, doi:10.1101/gr.181016.114 (2015).
20 Greer, S. U. *et al.* Genetic risk of cholangiocarcinoma is linked to the autophagy gene ATG7. *bioRxiv*, 836767, doi:10.1101/836767 (2019).
21 Imielinski, M. *et al.* Mapping the hallmarks of lung adenocarcinoma with massively parallel sequencing. *Cell* **150**, 1107-1120, doi:10.1016/j.cell.2012.08.029 (2012).
22 He, H. *et al.* Post-translational modifications of three members of the human MAP1LC3 family and detection of a novel type of modification for MAP1LC3B. *The Journal of biological chemistry* **278**, 29278-29287 (2003).
23 Liu, C. *et al.* Arginine68 is an essential residue for the C-terminal cleavage of human Atg8 family proteins. *BMC Cell Biol* **14**, 27, doi:10.1186/1471-2121-14-27 (2013).
24 Jatana, N., Ascher, D. B., Pires, D. E. V., Gokhale, R. S. & Thukral, L. Human LC3 and GABARAP subfamily members achieve functional specificity via specific structural modulations. *Autophagy* **16**, 239-255, doi:10.1080/15548627.2019.1606636 (2020).
25 Shintani, T. *et al.* Apg10p, a novel protein-conjugating enzyme essential for autophagy in yeast. *The EMBO journal* **18**, 5234-5241, doi:10.1093/emboj/18.19.5234 (1999).
26 Li, Q. *et al.* Burkholderia pseudomallei survival in lung epithelial cells benefits from miRNA-mediated suppression of ATG10. *Autophagy* **11**, 1293-1307, doi:10.1080/15548627.2015.1058474 (2015).
27 Jo, Y. K. *et al.* Polypyrimidine tract-binding protein 1-mediated down-regulation of ATG10 facilitates metastasis of colorectal cancer cells. *Cancer Lett* **385**, 21-27, doi:10.1016/j.canlet.2016.11.002 (2017).
28 Zhao, Q. *et al.* Dual Roles of Two Isoforms of Autophagy-related Gene ATG10 in HCV-Subgenomic replicon Mediated Autophagy Flux and Innate Immunity. *Scientific reports* **7**, 11250, doi:10.1038/s41598-017-11105-3 (2017).
29 Zhang, M. Q., Li, J. R., Peng, Z. G. & Zhang, J. P. Differential Effects of Autophagy-Related 10 Protein on HCV Replication and Autophagy Flux Are Mediated by Its Cysteine(44) and Cysteine(135). *Front Immunol* **9**, 2176, doi:10.3389/fimmu.2018.02176 (2018).
30 Gonzalez-Rodriguez, P. *et al.* SETD2 mutation in renal clear cell carcinoma suppress autophagy via regulation of ATG12. *Cell Death Dis* **11**, 69, doi:10.1038/s41419-020-2266-x (2020).
31 Ouyang, D. Y. *et al.* Autophagy is differentially induced in prostate cancer LNCaP, DU145 and PC-3 cells via distinct splicing profiles of ATG5. *Autophagy* **9**, 20-32, doi:10.4161/auto.22397 (2013).
32 Sharma, S. *et al.* A Phase II Clinical Trial of Oral Valproic Acid in Patients with Castration-Resistant Prostate Cancers Using an Intensive Biomarker Sampling Strategy. *Transl Oncol* **1**, 141-147, doi:10.1593/tlo.08136 (2008).
33 Wible, D. J., Chao, H. P., Tang, D. G. & Bratton, S. B. ATG5 cancer mutations and alternative mRNA splicing reveal a conjugation switch that regulates ATG12-ATG5-ATG16L1 complex assembly and autophagy. *Cell Discov* **5**, 42, doi:10.1038/s41421-019-0110-1 (2019).
34 Fletcher, K. *et al.* The WD40 domain of ATG16L1 is required for its non-canonical role in lipidation of LC3 at single membranes. *The EMBO journal* **37**, doi:10.15252/embj.201797840 (2018).
35 Itoh, T. *et al.* Golgi-resident small GTPase Rab33B interacts with Atg16L and modulates autophagosome formation. *Molecular biology of the cell* **19**, 2916-2925, doi:E07-12-1231, doi:10.1091/mbc.E07-12-1231 (2008).

36 Jiang, T., Qin, B., He, J., Lin, S. & Ding, S. Three isoforms of the Atg16L1 protein contribute different autophagic properties. *Mol Cell Biochem* **378**, 257-266, doi:10.1007/s11010-013-1616-8 (2013).
37 Lystad, A. H. *et al.* Distinct functions of ATG16L1 isoforms in membrane binding and LC3B lipidation in autophagy-related processes. *Nat Cell Biol* **21**, 372-383, doi:10.1038/s41556-019-0274-9 (2019).
38 Sadaghian Sadabad, M. *et al.* The ATG16L1-T300A allele impairs clearance of pathosymbionts in the inflamed ileal mucosa of Crohn's disease patients. *Gut* **64**, 1546-1552, doi:10.1136/gutjnl-2014-307289 (2015).
39 Gammoh, N. The multifaceted functions of ATG16L1 in autophagy and related processes. *J Cell Sci* **133**, doi:10.1242/jcs.249227 (2020).
40 Hampe, J. *et al.* A genome-wide association scan of nonsynonymous SNPs identifies a susceptibility variant for Crohn disease in ATG16L1. *Nat Genet* **39**, 207-211, doi:10.1038/ng1954 (2007).
41 Ishibashi, K. *et al.* Atg16L2, a novel isoform of mammalian Atg16L that is not essential for canonical autophagy despite forming an Atg12–5-16L2 complex. *Autophagy* **7**, 1500-1513, doi:10.4161/auto.7.12.18025 (2011).
42 Khor, B. *et al.* Distinct Tissue-Specific Roles for the Disease-Associated Autophagy Genes ATG16L2 and ATG16L1. *J Immunol* **203**, 1820-1829, doi:10.4049/jimmunol.1800419 (2019).
43 Cuervo, A. M. & Dice, J. F. A receptor for the selective uptake and degradation of proteins by lysosomes. *Science* **273**, 501-503 (1996).
44 Chi, C. *et al.* LAMP-2B regulates human cardiomyocyte function by mediating autophagosome-lysosome fusion. *Proc Natl Acad Sci U S A* **116**, 556-565, doi:10.1073/pnas.1808618116 (2019).
45 Fujiwara, Y. *et al.* Discovery of a novel type of autophagy targeting RNA. *Autophagy* **9**, 403-409, doi:10.4161/auto.23002 (2013).
46 Perez, L. *et al.* LAMP-2C Inhibits MHC Class II Presentation of Cytoplasmic Antigens by Disrupting Chaperone-Mediated Autophagy. *Journal of immunology* **196**, 2457-2465, doi:10.4049/jimmunol.1501476 (2016).
47 Wang, X. *et al.* PINK1 and Parkin target Miro for phosphorylation and degradation to arrest mitochondrial motility. *Cell* **147**, 893-906, doi:10.1016/j.cell.2011.10.018 (2011).
48 Safiulina, D. *et al.* Miro proteins prime mitochondria for Parkin translocation and mitophagy. *The EMBO journal* **38**, doi:10.15252/embj.201899384 (2019).
49 Marongiu, R. *et al.* Whole gene deletion and splicing mutations expand the PINK1 genotypic spectrum. *Hum Mutat* **28**, 98, doi:10.1002/humu.9472 (2007).
50 Sahashi, K. *et al.* In vitro and in silico analysis reveals an efficient algorithm to predict the splicing consequences of mutations at the 5' splice sites. *Nucleic Acids Res* **35**, 5995-6003, doi:10.1093/nar/gkm647 (2007).
51 Burns, M. P., Zhang, L., Rebeck, G. W., Querfurth, H. W. & Moussa, C. E. Parkin promotes intracellular Abeta1-42 clearance. *Hum Mol Genet* **18**, 3206-3216, doi:10.1093/hmg/ddp258 (2009).
52 Tan, M. M. X. *et al.* Genetic analysis of Mendelian mutations in a large UK population-based Parkinson's disease study. *Brain* **142**, 2828-2844, doi:10.1093/brain/awz191 (2019).
53 Khaminets, A. *et al.* Regulation of endoplasmic reticulum turnover by selective autophagy. *Nature* **522**, 354-358, doi:10.1038/nature14498 (2015).
54 Kohno, S., Shiozaki, Y., Keenan, A. L., Miyazaki-Anzai, S. & Miyazaki, M. An N-terminal-truncated isoform of FAM134B (FAM134B-2) regulates starvation-induced hepatic selective ER-phagy. *Life Sci Alliance* **2**, doi:10.26508/lsa.201900340 (2019).

55 Hu, X. *et al.* Transgenic mice overexpressing reticulon 3 develop neuritic abnormalities. *EMBO J* **26**, 2755-2767, doi:10.1038/sj.emboj.7601707 (2007).

56 Yang, Y. S. & Strittmatter, S. M. The reticulons: a family of proteins with diverse functions. *Genome Biol* **8**, 234, doi:10.1186/gb-2007-8-12-234 (2007).
